# Determination of Pyrrolizidine Alkaloids in Teas Using Liquid Chromatography–Tandem Mass Spectrometry Combined with Rapid-Easy Extraction

**DOI:** 10.3390/foods10102250

**Published:** 2021-09-23

**Authors:** Yujihn Kwon, Yongeui Koo, Yoonhwa Jeong

**Affiliations:** 1 Department of Food Science and Nutrition, Dankook University, 119 Dandaero, Cheonan-si 31116, Korea; tomato796@naver.com; 2 Food Contaminants Division, Food Safety Evaluation Department, National Food and Drug Safety Evaluation, Ministry of Food and Drug Safety, Osong, Cheongju 28159, Korea; csr502@daum.net

**Keywords:** pyrrolizidine alkaloids, tea, natural toxins, LC-MS/MS, SPE

## Abstract

This study developed an analytical method to determine pyrrolizidine alkaloids (PAs) in teas using liquid chromatography–tandem mass spectrometry combined with rapid-easy extraction. PAs were extracted with 40 mL of 0.05 M sulfuric acid in 50% methanol solution and cleaned up using Oasis MCX SPE cartridges. Chromatographic separation of 21 PAs was conducted on an X-Bridge C18 column with gradient elution. According to the AOAC official analysis methods, the developed method was verified to establish linearity, limits of detection, limits of quantification, accuracy, inter-day precision, and intra-day precision for each PA. Overall, the method showed excellent repeatability, sensitivity, and reproducibility. The verified method was applied to tea samples, including maté, lemon balm, fennel, hibiscus, chrysanthemum, lavender, oolong tea, chamomile, rooibos, peppermint, mix tea, black, and green tea. One of the main advantages of the method developed in this study is that it allows complete separation of lycopsamine and intermedine peaks. Therefore, the method could be used to monitor PAs in teas.

## 1. Introduction

Pyrrolizidine alkaloids (PAs) are natural toxins produced by plants for self-defense. These toxins are known to exist in Boraginaceae, Asteraceae, and Fabaceae plants that have pharmacological effects. However, caution is required when ingesting these due to toxins [1]. PAs are a class of alkaloids based on a pyrrolizidine structure, with more than 500 compounds found in more than 6000 plant species [1]. After PAs from plants are absorbed into the body, they are metabolized by the enzymes present in hepatocytes, become toxic, and excreted through the urine. PAs’ toxicity is associated with acute toxicity, which could result in the blockage of blood vessels and liver damage, chronic toxicity, and genotoxicity [2]. The 1,2-unsaturated PAs are predominantly toxic, and their toxicity level is highest for cyclic diesters, medium for non-cyclic diesters, and lowest for monoester structures [3,4,5]. Monocrotaline, retrorsine, senecionine, and integerrimine are cyclic diesters. However, heliotrine and heliotrine-N-oxide are non-cyclic diesters. The International Agency for Research on Cancer (IARC) classifies lasiocarpine, monocrotaline, and riddelliine as Group 2B (human carcinogens) and isatidine, retrorsine, seneciphylline, senkirkine, *Symphytum*, jacobine, and 18-hydroxysenkirkin as Group 3 [6,7].

Large amounts of PAs may be present in some teas due to the nature of the plant materials. In addition, teas may be contaminated with PAs from various plants (weeds) during growing and harvesting periods. In our study, the word “tea” indicates drinkable plant materials commonly referred to as tea by the general public or commercially by the food industry. According to the results of the quality inspection of infant tea products in the *German Oekotest* issue in May 2017, the amount of PAs in the two herb tea products containing fennel among the 18 products was higher than the tolerable daily intake (TDI: 0.007 µg/kg bw/day) recommended by Bundesinstitut für Risikobewertung (BfR) [8]. The European Food Safety Authority’s (EFSA) 2016 Activity Report on the emerging risks in the food hygiene sector included PAs in teas among the 17 potential new risks [9]. Around the world, many efforts have been taken to develop management methods to reduce the risks posed by PAs in teas and increase awareness about them. In South Korea, interest in the safety management of PAs continues to grow [10].

According to Korea Health Industry Development Institute’s report, the consumption of green tea by Koreans increased by 33% in 2013 (23.0 g) compared with 2010 (17.3 g) [11].

After signing free trade agreements (FTAs) with many countries, the volume of tea imports to South Korea increased steadily from approximately $3 million in 2009 to $11.15 million in 2014 [12]. In addition, the total tea production amount increased by 66.6% from KRW 492.2 billion in 2007 to KRW 819.7 billion in 2014 [13]. The exposure to PAs is expected to rise continuously due to the increased demand for well-being foods and food imports. Therefore, safety management of PAs in teas is necessary. However, the analysis of PAs is limited in the existing research data, and there are few cases of monitoring PAs in teas in South Korea. In our study, a PA analysis method was developed. Primary data were used for the analysis confirming the safety of teas distributed in South Korea.

The analysis of PAs is mainly based on high-performance liquid chromatography (HPLC), considering PAs’ physical and chemical properties [14]. Accurate and precise analytical methods using HPLC with mass spectrometry (MS) or tandem mass spectrometry MS/MS are commonly used in multi-component analysis [15,16,17]. Various purification methods such as thin-layer chromatography, column chromatography, liquid-liquid extraction (LLE), and solid-phase extraction (SPE) are applied to samples as pretreatment methods. Among them, SPE using a strong cation exchange stationary phase relying on the characteristics of tertiary amine groups of PAs is most widely used [18].

Research data on PAs reported in South Korea are limited [19,20]. BfR presented a method to determine 28 PAs in plant material that could be used to analyze PAs in tea [21]. However, BfR’s analytical method is limited because chromatograms between the compounds are not entirely separate. In our study, the sensitivity and separation of compounds are improved through liquid chromatography (LC) and rapid extraction and purification. The proposed method was validated, and the analysis of PAs in teas distributed in South Korea was performed to confirm the method’s practical applicability. According to the existing literature and intake level data, 290 tea samples belonging to 13 items were selected and analyzed. The results were compared with the results of previous studies.

## 2. Materials and Methods

### 2.1. Materials

Reference standards, including heliotrine, echimidine, europine, jacobine, lasiocarpine, lycopsamine, monocrotaline-N-oxide, senecionine-N-oxide, seneciphylline-N-oxide, senkirkine, and trichodesmine were purchased from Interpharm Corp. (Shanghai, China). Europine-N-oxide, heliotrine-N-oxide, intermedine, jacobine-N-oxide, lasiocarpine-N-oxide, monocrotaline, retrorsine, and retrorsine-N-oxide were purchased from PhytoLab (Vestenbergsgreuth, Germany), and senecionine and seneciphylline from Sigma-Aldrich Chemical Co. (St. Louis. Mo, USA). Acetonitrile and methanol were purchased from Merck Co. (HPLC grade, Darmstadt, Germany). Water was purified using the Barnstead NANO pure Diamond™ water purification system (Asheville, NC, USA). HPLC grade formic acid and ammonium formate were acquired from Sigma-Aldrich (St. Louis, MO, USA). In total, 290 tea samples belonging to 13 items (maté, lemon balm, fennel, hibiscus, chrysanthemum, lavender, oolong tea, chamomile, rooibos, peppermint, mixed tea, black tea, and green tea) were purchased offline at E-Mart and Lotte Mart, in Seoul, South Korea, and via South Korean internet stores (G-market, Auction, Coupang, and 11th Street) from March to September 2017. These 13 items were distributed commercially in South Korea, and previous studies detected PAs in them. The chrysanthemum tea was selected as a blank matrix, and preprocessing methods’ optimization and validation were performed. About 0.3–1.0 kg of samples were purchased depending on the number of edible portions. Edible portions were put together, homogenized, and kept in a freezer at −20 °C before the analysis.

### 2.2. Sample Preparation

Two grams of a homogenized sample were scaled into a 50 mL graduated polyethylene tube (Falcon, BD, Franklin Lakes, NJ, USA). The sample was extracted for 30 min by shaking with 40 mL of 0.05 M sulfuric acid in a 50% methanol solution. The extract was centrifuged for 10 min at 2900 G. After that, the supernatant was poured into a 50 mL tube and passed through a fluted filter paper (No.4, Whatman, Cambridge, UK). Before LC-MS/MS analysis, the filtrated crude extract (2 mL) was purified by SPE (SPE cartridge, Oasis MCX, 6 cc, 150 mg, Waters Corp, Dublin, Ireland).

The SPE cartridge had been previously conditioned with 3 mL of methanol and 3 mL of water. The 2 mL of crude extract was passed through the SPE cartridge at 2 mL/min. Then, the cartridge was washed with 4 mL of water and eluted with 4 mL of 2.5% ammonia in methanol. The eluted solution was dried using nitrogen gas and dissolved using 1 mL of 5% methanol. Finally, the resulting solution was filtered through a 0.22 μm PTFE chromacol syringe filter (Lab Unlimited Co., Dublin, Ireland) for LC-MS/MS analysis.

### 2.3. Preparation of Matrix-Matched Calibration Standards

PA standards dissolved in methanol or acetonitrile were used as stock solutions (1000 µg/mL). In total, 21 mixed stock solutions were prepared. The individual and mixed stock solutions were stored in a freezer (at −20 °C). Matrix-matched calibration standards were prepared by adding known amounts of mixed stock solutions to suitable volumes of the blank matrix extracts that were not contaminated with PAs. These blank matrix extracts were prepared by the same method as in the sample preparation section.

### 2.4. LC-MS/MS Analysis

Chromatographic analysis was conducted using a UPLC system (Nexera X2, Shimadzu Co., Tokyo, Japan). The injection volume of standards and the sample solution was 10 µL. The column (X-Bridge C18, 100 mm × 2.1 mm, 3.5 μm, water, Manchester, UK) was preserved at 40 °C. The mobile phase comprised two eluents, A (aqueous 5 mM ammonium formate and 0.1% formic acid) and B (95% methanol with 5 mM ammonium formate and 0.1% formic acid). The flow rate was set at 0.3 mL/min for all analyses. The elution was performed with the gradient at the following conditions: starting at 5% B for 0.5 min, increasing B from 5% to 30% for 6.5 min, from 30% to 95% for 4 min and then holding for 2 min, decreasing to 5% for 0.1 min, and finally holding for 1.9 min. The mass spectrometric analysis was performed using the LC-MS/MS system (Nexera X2 8060, Shimadzu Co., Tokyo, Japan) equipped with an electrospray ionization (ESI) source. The positive ion multiple reaction monitoring (MRM) mode was used to detect PAs. MS/MS conditions were optimized for the analysis of the toxins as follows: curtain gas (CUR), 25.0 psi; collision gas (CAD), 9 psi; ion spray voltage, 5.0 kV; ion source temperature, 350 °C; ion source gas (GS1), 50.0 psi; ion source gas (GS2), 50.0 psi; and source collision energy, 31–105 V (N_2_). The optimized MRM mode parameters for each PA are summarized in Table 1. Data processing was carried out using the Analyst software (Shimadzu, Tokyo, Japan).

### 2.5. Method Validation

The validation process was carried out according to the Association of Official Analytical Chemists (AOAC) official analysis methods [22]. The linearity, selectivity, repeatability, recovery, reproducibility, the matrix effects (ME) of the developed method, and the limit of quantification (LOQ) were evaluated. Twenty-one PAs were chosen as evaluated toxins. Selectivity was determined by the analysis of the blank tea samples, i.e., chrysanthemum. It was used as the blank matrix because PAs were not detected in the analysis, unlike other tea samples. The results identified any interfering peaks at the retention time of analytes using the multiple reaction monitoring (MRM) mode of two *m*/*z* transitions for each analyte.

Two calibration curves were produced: (1) a solvent standard calibration curve generated by diluting standard solutions with a solvent, and (2) a matrix-matched calibration curve obtained by spiking standard solutions to the extract of a blank sample. The peak areas of the PAs corresponding to their concentrations were plotted to construct calibration curves. The linearity of calibration curves was assessed by applying the least-squares method. The matrix effect (ME%) was evaluated by calculating the slope ratio of the matrix-matched calibration curve to the solvent standard calibration curve. The existence of signal suppression or signal enhancement (SSE) by the matrix could be inferred if the slope ratio is smaller or larger than 100%, respectively. An SSE < 50% and >150% suggests a strong matrix effect, an SSE 50–80% or 120–150% indicates a medium matrix effect, and an SSE 80–120% points to a low matrix effect [23].

Accuracy was measured by analyzing the blank samples spiked at three concentration levels (low, medium, and high). The analysis was replicated three times at each concentration level. Two kinds of precision were investigated: repeatability for inter-day precision and reproducibility for intra-day precision. Inter-day precision was examined on three different days. Precision values were expressed as the relative standard deviation (%RSD), and accuracy values were determined by the recovery method (%). A mixed-standard solution was added to each blank sample and analyzed under optimized conditions. The lowest detectable concentration (LOD) had a signal-to-noise ratio of at least 3. The lowest quantifiable concentration (LOQ) had a signal-to-noise ratio of at least 10.

## 3. Results and Discussion

### 3.1. Sample Preparation

**Figure 1:**
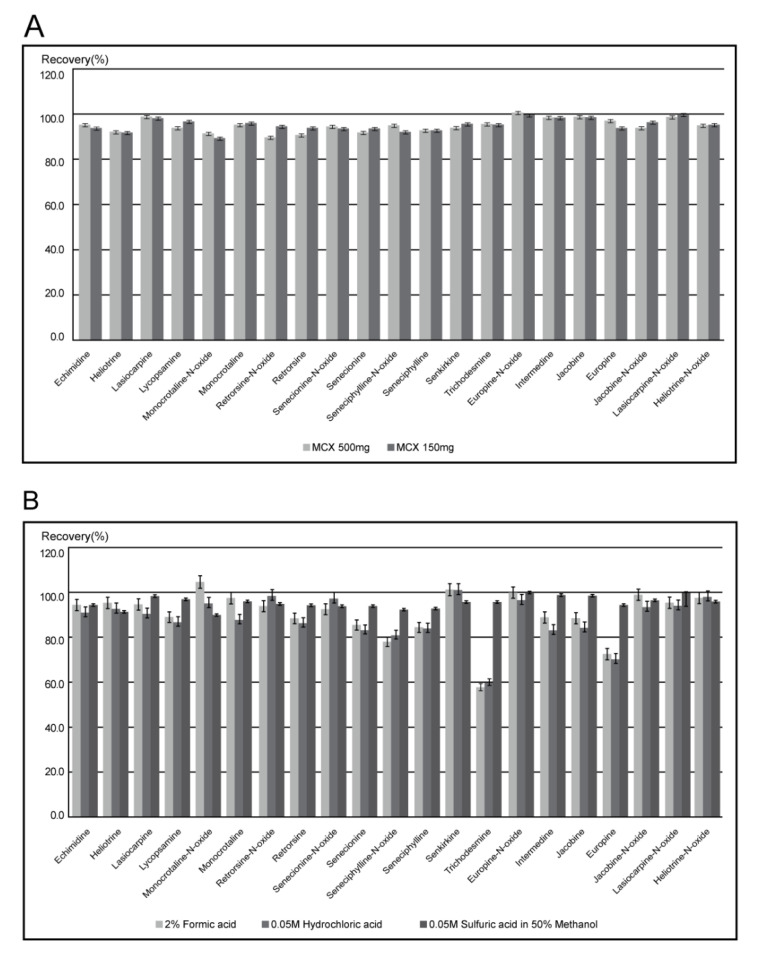
Recovery rates of PAs in tea (chrysanthemum) depending on (**A**) cartridge and (**B**) extraction solvent.

The extraction solvents, including 0.05 M sulfuric acid [21,24], 0.05 M hydrochloric acid [25], and 2% formic acid in water [26], are mainly used for the analysis of PAs. The recovery rate using the solvent of 0.05 M sulfuric acid in 50% methanol was compared with those in other studies to select a highly efficient solvent for extracting PAs with low impurities. The recovery rates using three extraction solvents in tea samples are shown in Figure 1b. In 14 PAs, including echimidine, the recovery rates were similar for the three extraction solvents. On the other hand, for senecionine, seneciphylline-N-oxide, seneciphylline, trichodesmine, intermedine, jacobine, and europine, the recovery rates increased to 40% when 0.05 M sulfuric acid in 50% methanol was used as the extraction solvent. This solvent was selected since 0.05 M sulfuric acid in 50% methanol yielded relatively high recovery rates and was further optimized in this study.

In other studies, purification of PAs was carried out using SPE cartridges such as MCX, Strata-X, SCX, and DSC-C18 cartridges [19,21,24,25]. In our study, the four SPE cartridges used in other studies were also tested to purify PAs in tea samples. DSC-C18 and Strata-X cartridges are hydrophobic silica-based sorbents with wide pore sizes. They are the most widely used sorbents with a high affinity for non-polar compounds (max 75 kD). Mixed-mode cation exchange (MCX) cartridges are strong acid cation exchange resins, including the sulfuric acid groups. Their properties are useful for separating components with pKa < 1. The MCX cartridges show a relatively high recovery rate and good repeatability due to these properties. Although the MCX cartridge with 500 mg capacity showed a similar recovery rate to the cartridge with 150 mg capacity, the cartridge with 150 mg capacity was selected due to a shorter purification time (2 h) than that with the 500 mg capacity (Figure 1a).

**Figure 2:**
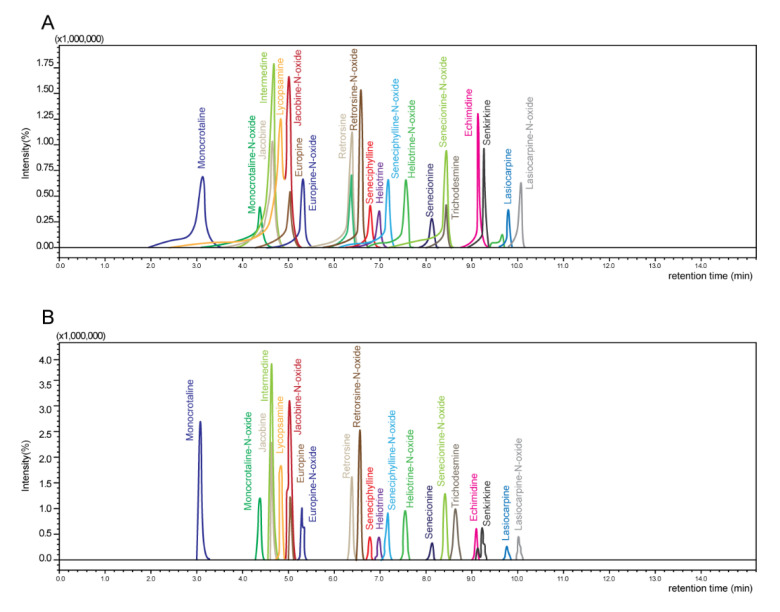
Comparison of sensitivity for reconstitution solvents between (**A**) 100% methanol and (**B**) 5% methanol.

In our study, the 5% and 100% methanol were tested as reconstitution solvents because these were used in previous research [19,21,24,25]. The 5% methanol showed about 2 to 3 times higher sensitivity than 100% methanol, and the tailing of the measured peaks decreased significantly when 5% methanol was used (Figure 2). As a result, 5% methanol was selected as a reconstitution solvent.

### 3.2. Optimum Conditions of LC-MS/MS Analysis

The buffer solutions, including formic acid and ammonium formate, were mainly used for reversed-phase HPLC separation in previous research [18,21,24]. Formic acid is a common additive constituent of the aqueous mobile phase in reversed-phase liquid chromatography-mass spectrometry (LC-MS). Ammonium formate and formic acid were used as proton sources in the positive ionization mode for the LC-MS analysis by producing [M + H]^+^ and [M + NH4]^+^. The formic acid and ammonium formate concentration in the aqueous mobile phase was optimized using the test results of response intensity, repeatability, and chromatogram shape. As a result, it was confirmed that the solution containing 5 mM ammonium formate and 0.1% formic acid was suitable as the mobile phase for PAs.

**Figure 3:**
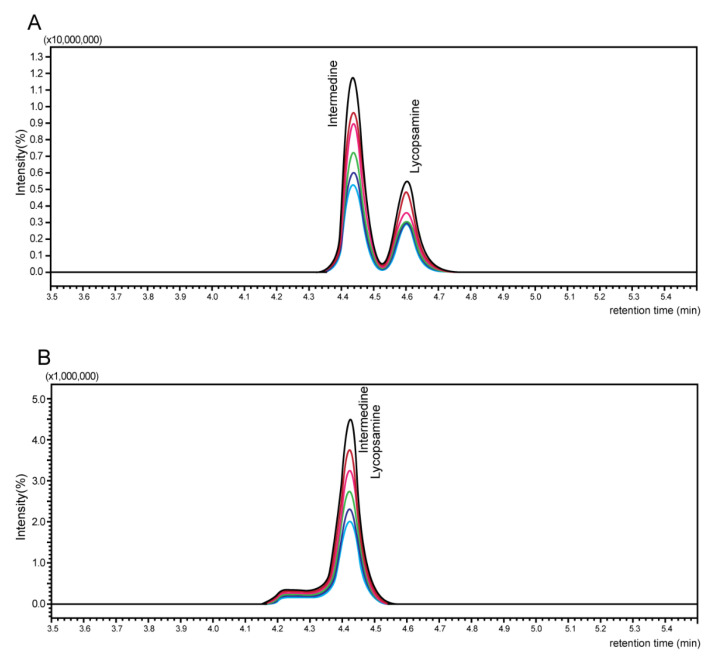
Comparison of chromatogram using mobile phase solvents (**A**) 95% methanol and (**B**) 100% methanol.

In addition, a mobile phase comparison experiment was performed to find a suitable mobile phase for the analysis of 21 PAs. In the PA analysis, 100% methanol solution containing 5 mM ammonium formate and 0.1% formic acid solution and 95% methanol solution containing 5 mM ammonium formate and 0.1% formic acid solution were compared as the mobile phase B for peak separation of isomeric lycopsamine and intermedine. In the BfR method, when 100% methanol solution containing 5 mM ammonium formate and 0.1% formic acid solution was used as the mobile phase B solvent, there was a problem with peaks not being completely separate. As a result of the comparison, 95% methanol solution containing 5 mM ammonium formate and 0.1% formic acid solution was finally selected because the two peaks were entirely separate when the solution was used as the mobile phase B solvent (Figure 3). When the test for column flow rate was conducted in the range of 50 to 300 μL/min, the best result was obtained at 300 μL/min under suitable conditions for mass spectrometry. For column oven temperature, the best result was obtained at 40 °C as a result of evaluating the range of 20 to 40 °C. In addition, the injection volumes were examined in the range of 5–20 μL, and the best result was obtained at 10 μL. Overall, the 21 PAs were separated successfully, and any interfering peaks were not observed around the toxins.

The MS/MS conditions were checked by reviewing the previously reported reference [21]. It was confirmed that the parent and daughter ions’ pattern in our study appeared similar to the reference. The MS parameters were optimized to improve the selectivity and sensitivity of the analytical method. The PAs were measured in the MRM acquisition mode by observing two transitions for each parent ion. Consequently, the most potent fragments of each toxin were used as quantification ions, whereas the other fragments were used as confirmation ions.

Based on the PAs assay recommended by BfR [21], this analytical method was established by optimizing each parameter by considering the characteristics of the sample matrix and the material properties of PAs. One of the main advantages of our method is that the peaks of isomers, lycopsamine, and intermedine were separated entirely in the analysis of 21 PAs. Moreover, compared with the analytical method suggested by BfR, our study’s approach enabled quicker and more effective extraction and purification.

### 3.3. Method Validation

The validation process was conducted according to the AOAC official methods of analysis [22]. The method’s selectivity was determined by the absence of interfering peaks of target analytes in the blank tea samples, i.e., chrysanthemum tea. Table 2 summarizes the results for LOQ, LOD, ME, linearity, and range. For all analytes, the matrix-matched calibration curves showed good linearity (R^2^ > 0.997). The LOD (S/N = 3) and LOQ values (S/N = 10) were 0.1–3.0 μg/kg and 0.3–9.0 μg/kg for each PA in the tea samples. These LOD and LOQ values were similar to those recommended by BfR [21]. The matrix-matched calibration curves were applied to obtain reliable results. Ionization suppression or enhancement was demonstrated by comparing the slopes of the matrix-matched standard calibration curve and solvent standard one. It was reported that the ME was higher for PAs in teas, and the ionic inhibition effect was usually observed [26].

In our study, PAs showed medium and strong inhibition of ME in the tea samples. Thus, it was thought that matrix-matched calibration curves must be applied to analyze PAs in tea samples. Table 3 shows the results for the average recovery rate and precision of the developed method. This method’s recovery rate and precision were evaluated for each toxin at three levels (2 LOQ, 5 LOQ, and 10 LOQ) of concentration for 3 days with three replications. The PAs’ average recovery rates ranged from 86.72% to 101.44%, similar to the recovery rates of AOAC (from 50% to 120%). Precision values were expressed as within laboratory reproducibility (inter-day) and repeatability (intra-day). These were allowable based on the AOAC criteria of RSD 20%. The intra-day precision rates (%RSD) ranged from 0.08% to 3.88%. The inter-day precision rates (%RSD) ranged from 0.5% to 4.82%.

### 3.4. Application for Commercial Tea Samples

The method developed in this study was applied to determine 21 PAs in 290 commercial tea samples, including maté, lemon balm, fennel tea, hibiscus, chrysanthemum, lavender, oolong tea, chamomile, rooibos, peppermint, mix tea, black, and green tea obtained from different regions in South Korea. The detailed data for 290 commercial tea samples are listed in the supplementary materials (Tables S1–S13).As the analysis of the commercial tea samples (Table 4) demonstrates, 62 samples were found to be above LOQ. The highest detection rate was in rooibos tea, and the average amount of PAs in rooibos tea was 0.17 mg/kg. That was followed by lemon balm (0.50 mg/kg), peppermint (0.37 mg/kg), and herbal mix tea (0.37 mg/kg). Mulder et al. [27] investigated PAs in teas produced in Western European countries, such as France, Germany, and Spain. They found that the average amounts of PAs were 454.1 μg/kg for rooibos tea, 496.2 μg/kg for peppermint tea, 273.8 μg/kg for chamomile tea, 439.4 μg/kg for mixed tea, 555.8 μg/kg for black, and 447.5 μg/kg for green tea [27]. Mulder et al.’s results are similar to the results of our study, except for green tea. In our study, PAs were not detected in Korean green tea. Green tea is brewed from Camellia sinensis leaves worldwide, but the amount of PAs in green tea differs depending on the region. It is thought that PAs were detected in green tea because it was contaminated by weeds containing large amounts of PAs and not because green tea leaves produced PAs. Jank et al. [28] also reported that the cause of PAs’ detection in green tea was weed contamination.

Out of 21 PAs, 15 were detected, including echimidine, heliotrine, lasiocarpine, lycopsamine, retrosine-N-oxide, senecionine-N-oxide, senecionine, seneciphylline-N-oxide, seneciphylline, senkirkine, trichodesmine, europine-N-oxide, intermedine, lasiocarpine-N-oxide, and heliotrine-N-oxide (Table 5). Senecionine-N-oxide was detected most often: in 29 out of 290 samples. It was followed by senecionine, which was detected in 27 samples. These results were similar to the results obtained for tea by Bodi et al. [18]. The results confirm that raw materials for tea could be contaminated with PA-containing weeds. Europine-N-oxide showed the highest concentration (0.74 mg/kg), followed by seneciphylline-N-oxide (0.53 mg/kg), and lasiocarpine (0.40 mg/kg).

## 4. Conclusions

The effectiveness of liquid chromatography (LC)–mass spectrometry (MS/MS) analysis was confirmed based on the verification criteria such as specificity, linearity, precision, accuracy, recovery rate, detection, and quantitation limits set out by AOAC official methods of analysis. The method proposed in this study was successfully validated and applied to the tea samples containing maté, lemon balm, fennel, hibiscus, chrysanthemum, lavender, oolong tea, chamomile, rooibos, peppermint, mix tea, black, and green tea. The average amounts of PAs in teas were relatively high in lemon balm, peppermint, and mixed teas, in which senecionine and senecionine N-oxide were mainly detected. Therefore, it was concluded that teas imported to South Korea could be contaminated by weeds or plants of the genus *Senecio.* The method developed in our study could be used to monitor PAs in tea.

## Figures and Tables

**Table 1:** Optimum parameters of MRM mode depending on PA analyte.

Analytes	M.W. (g/mol)	Precursor Ion (*m*/*z*)	Product Ion (*m*/*z*)	Dwell Time (msec)	Q1 (volts)	CE*a* (volts)	Q3 (volts)
Echimedine	397.47	398.25	120.10 ^b^	4	−11	−25	−24
			220.10	4	−11	−17	−25
Heliotrine	313.39	314.20	138.15 ^b^	4	−11	−20	−28
			156.20	4	−11	−28	−17
Lasiocarpine	411.49	412.20	120.10 ^b^	4	−15	−28	−24
			336.15	4	−14	−19	−24
Lycopsamine	299.37	300.20	94.10 ^b^	4	−11	−25	−18
			138.10	4	−11	−20	−24
Monocrotaline	325.40	326.15	120.10 ^b^	4	−12	−35	−22
			94.10	4	−12	−47	−17
Monocrotaline-N-oxide	341.36	342.15	137.10 ^b^	4	−12	−29	−14
			119.10	4	−12	−31	−22
Retrorsine-N-oxide	367.40	368.20	94.20 ^b^	4	−13	−49	−19
			118.05	4	−13	−32	−21
Retrorsine	351.40	352.20	120.10 ^b^	4	−12	−28	−13
			138.15	4	−12	−30	−29
Senecionine-N-oxide	351.17	352.20	94.10 ^b^	4	−12	−47	−19
			118.05	4	−12	−30	−24
Senecionine	335.39	336.20	120.10 ^a^	4	−12	−28	−21
			94.05	4	−12	−35	−18
Seneciphylline-N-oxide	349.40	350.20	94.10 ^b^	4	−12	−43	−20
			120.15	4	−12	−34	−24
Seneciphylline	333.40	334.15	120.10 ^b^	4	−12	−28	−22
			94.10	4	−12	−34	−17
Senkirkine	365.42	366.20	168.15 ^b^	4	−10	−30	−18
			122.15	4	−13	−33	−22
Trichodesmone	353.41	354.15	189.20 ^b^	4	−12	−29	−20
			149.10	4	−12	−25	−30
Europine-N-oxide	345.39	346.30	172.05 ^b^	4	−17	−31	−18
			111.10	4	−17	−44	−12
Intermedine	299.37	300.30	94.15 ^b^	4	−15	−27	−19
			138.05	4	−15	−20	−14
Jacobine	351.40	352.30	120.15 ^b^	4	−17	−31	−22
			155.15	4	−17	−29	−16
Europine	329.39	330.10	181.15 ^b^	4	−16	−34	−19
			239.10	4	−16	−25	−26
Jacobine-N-oxide	367.39	368.10	296.15 ^b^	4	−18	−26	−20
			120.15	4	−13	−38	−12
Lasiocarpine-N-oxide	427.50	428.20	254.30 ^b^	4	−15	−29	−28
			93.85	4	−10	−48	−10
Heliotrine-N-oxide	329.39	330.10	172.15 ^b^	4	−12	−27	−18
			111.10	4	−12	−43	−20

Note. ^a^ quantification ion, ^b^ collision energy (CE).

**Table 2:** LOD, LOQ, matrix effects, and calibration curves of the PAs in tea (chrysanthemum).

Analytes	RT (min)	LOD (μg/kg)	LOQ (μg/kg)	Matrix Effect (%)	Range (μg/kg)	Slope	Intercept	R^2^
Echimidine	9.01	0.1	0.3	31.9	0.20–6.00	4.19 × 10^5^	−3300.44	0.9997
Heliotrine	6.87	0.2	0.6	70.8	0.30–12.00	4.50 × 10^5^	12,495.3	0.9999
Lasiocarpine	9.64	0.8	2.4	47.6	1.20–48.00	5.77× 10^4^	3418.49	0.9998
Lycopasamine	4.96	0.3	0.9	72.6	0.50–18.00	1.62 × 10^6^	37,066.4	0.9998
Monocrotaline -N-oxide	4.32	1.0	2.9	66.7	1.50–58.00	2.00 × 10^5^	9351.2	0.9996
Monocrotaline	3.05	1.3	3.8	56.3	1.90–76.00	3.62 × 10^5^	133,155	0.9999
Retrorsine -N-oxide	6.46	3.0	9.0	89.7	4.50–180.00	8.96 × 10^4^	241,711	0.9987
Retrorsine	6.29	2.1	6.4	83.9	3.20–128.00	9.82 × 10^4^	31,222.1	0.9998
Senecionine -N-oxide	8.29	0.5	1.5	76.5	0.80–30.00	3.69 × 10^5^	129,756	0.9991
Senecionine	8.01	0.6	1.8	60.1	0.90–36.00	8.25 × 10^4^	−6769.52	0.9998
Seneciphylline -N-oxide	7.05	1.3	3.9	68.5	2.00–78.00	9.33 × 10^4^	26,725.2	0.9999
Seneciphylline	6.67	0.9	2.8	61.2	1.40–56.00	7.56 × 10^4^	−2383.93	0.9998
Senkirkine	9.11	0.3	0.8	44.4	0.40–16.00	4.44 × 10^5^	−30,200.6	0.9999
Trichodesmine	8.97	0.3	0.9	50.6	0.50–18.00	3.10 × 10^5^	−6440.8	0.9998
Europine -N-oxide	5.24	0.8	2.3	94.3	1.20–46.00	2.67 × 10^5^	79,617.5	0.9996
Intermedine	4.77	0.3	1.0	73.1	0.50–20.00	7.14 × 10^5^	−22,329.7	0.9999
Jacobine	4.57	1.7	5.1	67.8	2.60–102.00	2.89 × 10^5^	127,995	0.9995
Europine	5.23	2.3	6.9	68.4	3.50–138.00	6.23 × 10^4^	110,903	0.9989
Jacobine -N-oxide	4.99	0.9	2.7	68.4	1.40–54.00	1.34 × 10^5^	26,550	0.9998
Lasiocarpine -N-oxide	9.89	0.3	0.8	52.6	0.40–16.00	2.27 × 10^5^	−790.995	0.9999
Heliotrine -N-oxide	7.44	0.1	0.4	79.1	0.20–6.40	1.70 × 10^6^	−72,287.8	0.9994

**Table 3:** Accuracy and precision (n = 3) for the developed LC-MS/MS method in tea (chrysanthemum).

Analyte	Conc. (μg/kg)	Recovery (RSD%)		Analyte	Conc. (μg/kg)	Recovery (RSD%)		Analyte	Conc. (μg/kg)	Recovery (RSD%)	
		Intra-day	Inter-day			Intra-day	Inter-day			Intra-day	Inter-day
Echimidine	0.6	90.45 (1.96)	95.83 (1.05)	Retrorsine	12.8	92.96 (3.88)	95.25 (4.59)	Europine-N-oxide	4.6	96.65 (2.05)	96.47 (4.55)
	1.5	96.61 (2.45)	97.01 (3.51)		32.0	93.81 (0.48)	96.63 (2.99)		11.4	101.44 (0.48)	98.30 (2.77)
	3.1	95.12 (2.81)	96.74 (1.62)		64.0	95.44 (0.08)	97.01 (2.92)		22.8	101.02 (1.06)	100.41 (2.87)
Heliotrine	1.2	86.72 (3.09)	97.38 (0.74)	Senecionine-N-oxide	2.9	94.73 (1.76)	95.86 (2.39)	Intermedine	1.9	99.24 (0.84)	97.96 (1.07)
	3.0	94.73 (2.13)	97.65 (1.20)		7.3	92.44 (1.09)	97.38 (4.14)		4.9	99.02 (1.46)	96.26 (2.58)
	6.0	94.96 (1.59)	97.48 (2.49)		14.6	94.39 (0.99)	96.81 (1.26)		9.7	97.65 (0.59)	97.82 (1.16)
Lasiocarpine	4.8	99.09 (0.78)	96.25 (4.01)	Senecionine	3.7	88.19 (2.88)	95.72 (2.32)	Jacobine	10.3	97.75 (1.86)	97.94 (0.95)
	11.8	96.47 (2.15)	96.47 (1.58)		9.2	91.52 (2.27)	92.71 (2.06)		25.7	100.52 (1.58)	97.49 (0.37)
	23.6	99.52 (1.88)	97.32 (1.12)		18.5	97.06 (0.98)	97.86 (1.75)		51.3	97.35 (1.19)	99.89 (2.13)
Lycopasamine	1.8	95.53 (0.88)	97.23 (0.96)	Seneciphylline-N-oxide	7.9	90.05 (2.26)	96.94 (4.44)	Europine	13.8	95.20 (1.70)	95.83 (3.55)
	4.5	97.82 (1.46)	98.16 (1.09)		19.7	91.90 (1.96)	96.16 (4.32)		34.5	94.92 (2.52)	96.37 (2.41)
	9.0	97.02 (0.86)	98.26 (0.55)		39.4	94.65 (1.16)	97.47 (3.09)		68.9	92.75 (1.63)	98.00 (1.49)
Monocrotaline-N-oxide	5.9	91.03 (0.56)	93.76 (1.12)	Seneciphylline	5.6	94.31 (0.71)	91.64 (0.96)	Jacobine-N-oxide	5.4	94.03 (2.77)	97.36 (0.90)
	14.7	88.90 (1.09)	96.42 (2.14)		14.0	90.14 (2.15)	94.50 (2.85)		13.5	97.43 (0.32)	97.15 (0.88)
	29.4	88.94 (1.19)	94.98 (1.01)		28.1	94.83 (0.41)	96.06 (0.41)		27.0	97.84 (1.82)	99.12 (1.49)
Monocrotaline	7.7	92.47 (1.84)	94.24 (2.36)	Senkirkine	1.5	95.35 (1.52)	97.91 (3.68)	Lasiocarpine -N-oxide	1.5	101.40 (3.54)	96.36 (1.94)
	19.2	96.34 (0.78)	95.86 (1.24)		3.8	97.64 (1.24)	96.59 (0.92)		3.8	99.29 (2.31)	100.04 (4.18)
	38.4	99.63 (0.65)	96.41 (3.26)		7.6	94.12 (0.64)	97.17 (2.23)		7.7	98.75 (2.10)	101.32 (3.35)
Retrorsine -N-oxide	18.0	92.61 (0.70)	96.33 (2.76)	Trichodesmine	1.8	96.95 (3.49)	95.17 (2.22)	Heliotrine-N-oxide	0.7	92.29 (1.28)	96.56 (4.82)
	45.0	96.28 (0.63)	94.83 (2.72)		4.4	93.54 (1.14)	95.22 (1.02)		1.8	96.29 (1.23)	96.62 (0.15)
	90.1	95.09 (0.90)	96.89 (2.10)		8.8	96.55 (1.10)	97.24 (3.21)		3.6	98.47 (0.96)	98.89 (2.13)

**Table 4:** Total PA concentration in teas.

Tea Type	*n* > LOD / *n*	Mean (mg/kg)	Minimum (mg/kg)	Maximum (mg/kg)
Rooibos	18/23	0.17	0.02	0.67
Peppermint	11/25	0.37	0.01	1.23
Lavender	8/20	0.08	0.002	0.22
Chamomile	8/21	0.07	0.02	0.12
Lemon balm	6/18	0.50	0.06	1.88
Mix tea	7/25	0.37	0.01	1.49
Black tea	3/31	0.12	0.07	0.16
Maté	1/16	0.04	0.04	0.04
Green tea	0/32	-	-	-
Oolong tea	0/21	-	-	-
Chrysanthemum	0/20	-	-	-
Fennel	0/19	-	-	-
Hibiscus	0/19	-	-	-
Total	62/290	0.23	0.002	1.88

**Table 5:** Summary of targeted PAs detected.

PA	Number of Samples (N = 290)	Mean Concentration (mg/kg)	Minimum Concentration (mg/kg)	Maximum Concentration (mg/kg)
Echimidine	8	0.04	0.003	0.16
Heliotrine	13	0.03	0.01	0.11
Lasiocarpine	13	0.11	0.02	0.40
Lycopsamine	1	0.01	0.01	0.01
Retrorsine-N-oxide	6	0.11	0.05	0.18
Senecionine-N-oxide	29	0.09	0.01	0.36
Senecionine	27	0.08	0.02	0.30
Seneciphylline-N-oxide	7	0.20	0.01	0.53
Seneciphylline	4	0.11	0.05	0.17
Senkirkine	2	0.01	0.01	0.01
Trichodesmine	20	0.04	0.01	0.20
Europine-N-oxide	14	0.18	0.06	0.74
Intermedine	3	0.04	0.02	0.07
Lasiocarpine-N-oxide	12	0.07	0.01	0.35
Heliotrine-N-oxide	14	0.06	0.004	0.29

## Data Availability

The datasets generated for this study are available on request to the corresponding author.

## Supplementary Materials

The following are available online at https://www.mdpi.com/article/10.3390/foods10102250/s1, Table S1: Levels of pyrrolizidine alkaloids in rooibos, Table S2: Levels of pyrrolizidine alkaloids in peppermint, Table S3: Levels of pyrrolizidine alkaloids in lavender, Table S4: Levels of pyrrolizidine alkaloids in chamomile, Table S5: Levels of pyrrolizidine alkaloids in lemon balm, Table S6: Levels of pyrrolizidine alkaloids in mix tea, Table S7: Levels of pyrrolizidine alkaloids in black tea, Table S8: Levels of pyrrolizidine alkaloids in maté, Table S9: Levels of pyrrolizidine alkaloids in green tea, Table S10: Levels of pyrrolizidine alkaloids in oolong tea, Table S11: Levels of pyrrolizidine alkaloids in chrysanthemum Table S12: Levels of pyrrolizidine alkaloids in fennel Table S13: Levels of pyrrolizidine alkaloids in hibiscus..
