# Supplementary material for: Determination of Pyrrolizidine Alkaloids in Teas Using Liquid Chromatography–Tandem Mass Spectrometry Combined with Rapid-Easy Extraction
Source: Foods. 2021 Sep 23;10(10):2250. doi: 10.3390/foods10102250 (PMC8534422; doi:10.3390/foods10102250)
Supplement: Supplementary file 1 [file foods-10-02250-s001.zip › foods-1357806-supplementary.pdf]

Table S1. Rooibos

	1	2	3	4	5	6	7	8	9	10	11	12	13	14	15	16	17	18	19	20	21	T O T A L (mg/kg)
S A1	N.D	N.D	N.D	N.D	N.D	N.D	<LOQ	0	0.36	0.30	N.D	N.D	<LOQ	N.D	N.D	N.D	N.D	N.D	N.D	N.D	N.D	0.67
S A2	N.D	N.D	N.D	N.D	N.D	N.D	0.12	<LOQ	0.21	0.15	N.D	N.D	0.01	N.D	N.D	N.D	N.D	N.D	N.D	N.D	N.D	0.49
S A3	N.D	N.D	N.D	N.D	N.D	N.D	<LOQ	<LOQ	0.11	0.12	N.D	N.D	<LOQ	N.D	N.D	N.D	N.D	N.D	N.D	N.D	N.D	0.24
S A4	N.D	N.D	N.D	N.D	N.D	N.D	<LOQ	N.D	0.13	0.09	N.D	N.D	<LOQ	N.D	N.D	N.D	N.D	N.D	N.D	N.D	N.D	0.22
S A5	N.D	N.D	N.D	N.D	N.D	N.D	N.D	N.D	0.11	0.08	N.D	N.D	<LOQ	N.D	N.D	N.D	N.D	N.D	N.D	N.D	N.D	0.19
S A6	N.D	N.D	N.D	N.D	N.D	N.D	<LOQ	N.D	0.11	0.06	N.D	N.D	0.01	N.D	N.D	<LOQ	N.D	N.D	N.D	N.D	N.D	0.18
S A7	<LOQ	N.D	N.D	N.D	N.D	N.D	N.D	N.D	0.09	0.09	N.D	N.D	<LOQ	N.D	N.D	N.D	N.D	N.D	N.D	N.D	N.D	0.18
S A8	N.D	N.D	N.D	N.D	N.D	N.D	<LOQ	N.D	0.06	0.06	N.D	N.D	<LOQ	N.D	N.D	N.D	N.D	N.D	N.D	N.D	N.D	0.12
S A9	0.02	<LOQ	N.D	N.D	N.D	N.D	N.D	N.D	0.03	0.07	N.D	<LOQ	<LOQ	N.D	N.D	N.D	N.D	N.D	N.D	N.D	<LOQ	0.12
S A10	N.D	N.D	N.D	N.D	N.D	N.D	<LOQ	N.D	0.06	0.06	N.D	N.D	<LOQ	N.D	N.D	<LOQ	N.D	N.D	N.D	<LOQ	<LOQ	0.12
S A11	<LOQ	N.D	N.D	N.D	N.D	N.D	N.D	N.D	0.05	0.04	N.D	N.D	<LOQ	N.D	N.D	N.D	N.D	N.D	N.D	N.D	N.D	0.08
S A12	N.D	N.D	N.D	N.D	N.D	N.D	<LOQ	N.D	0.04	0.04	N.D	N.D	<LOQ	N.D	N.D	N.D	N.D	N.D	N.D	N.D	N.D	0.07
S A13	<LOQ	N.D	N.D	N.D	N.D	N.D	N.D	N.D	0.04	0.03	N.D	N.D	<LOQ	N.D	N.D	<LOQ	N.D	N.D	N.D	N.D	N.D	0.07
S A14	N.D	N.D	N.D	N.D	N.D	N.D	N.D	N.D	0.02	0.04	N.D	N.D	<LOQ	N.D	N.D	N.D	N.D	N.D	N.D	N.D	N.D	0.07
S A15	N.D	N.D	N.D	N.D	N.D	N.D	N.D	N.D	0.03	0.04	N.D	N.D	<LOQ	N.D	N.D	N.D	N.D	N.D	N.D	N.D	N.D	0.06
S A16	<LOQ	N.D	N.D	N.D	N.D	N.D	N.D	N.D	0.02	0.02	N.D	N.D	<LOQ	N.D	N.D	<LOQ	N.D	N.D	N.D	N.D	N.D	0.04
S A17	N.D	N.D	N.D	N.D	N.D	N.D	N.D	N.D	0.02	0.02	N.D	N.D	<LOQ	N.D	N.D	N.D	N.D	N.D	N.D	N.D	N.D	0.04
S A18	0.01	N.D	N.D	N.D	N.D	N.D	N.D	N.D	0.01	<LOQ	N.D	N.D	N.D	N.D	N.D	N.D	N.D	N.D	N.D	N.D	N.D	0.02
S A19	N.D	N.D	N.D	N.D	N.D	N.D	N.D	N.D	N.D	<LOQ	N.D	N.D	N.D	N.D	N.D	N.D	N.D	N.D	N.D	N.D	N.D	–
S A20	N.D	N.D	N.D	N.D	N.D	N.D	N.D	N.D	N.D	N.D	N.D	N.D	N.D	N.D	N.D	N.D	N.D	N.D	N.D	N.D	N.D	–
S A21	N.D	N.D	N.D	N.D	N.D	N.D	N.D	N.D	N.D	N.D	N.D	N.D	N.D	N.D	N.D	N.D	N.D	N.D	N.D	N.D	N.D	–
S A22	<LOQ	N.D	N.D	N.D	N.D	N.D	N.D	N.D	N.D	N.D	N.D	N.D	N.D	N.D	N.D	N.D	N.D	N.D	N.D	N.D	N.D	–

S A23	N.D	N.D	N.D	N.D	N.D	N.D	N.D	N.D	N.D	N.D	N.D	N.D	N.D	N.D	N.D	N.D	N.D	N.D	N.D	N.D	N.D	–

Table S2. Peppermint tea

	1	2	3	4	5	6	7	8	9	10	11	12	13	14	15	16	17	18	19	20	21	T O T A L (mg/kg)
S A1	N.D	N.D	N.D	N.D	N.D	N.D	0.09	<LOQ	0.25	0.24	0.48	0.17	N.D	N.D	N.D	N.D	N.D	N.D	N.D	N.D	N.D	1.23
S A2	<LOQ	0.04	0.11	N.D	N.D	N.D	N.D	N.D	N.D	N.D	N.D	N.D	N.D	N.D	0.29	N.D	N.D	N.D	N.D	0.06	0.09	0.59
S A3	N.D	0.03	0.11	N.D	N.D	N.D	N.D	N.D	N.D	N.D	N.D	N.D	N.D	<LOQ	0.24	N.D	N.D	N.D	N.D	0.09	0.10	0.57
S A4	N.D	<LOQ	N.D	N.D	N.D	N.D	<LOQ	N.D	0.07	0.07	0.14	0.11	N.D	0.03	<LOQ	N.D	N.D	N.D	N.D	<LOQ	0.004	0.42
S A5	N.D	<LOQ	<LOQ	N.D	N.D	N.D	0.08	N.D	0.06	0.05	0.10	0.05	N.D	0.02	<LOQ	N.D	N.D	N.D	N.D	<LOQ	0.005	0.37
S A6	N.D	0.01	0.08	N.D	N.D	N.D	N.D	N.D	N.D	N.D	N.D	N.D	N.D	N.D	0.16	N.D	N.D	N.D	N.D	0.04	0.06	0.36
S A7	N.D	0.02	0.06	N.D	N.D	N.D	N.D	N.D	N.D	N.D	N.D	N.D	N.D	N.D	0.08	N.D	N.D	N.D	N.D	0.04	0.03	0.22
S A8	N.D	0.01	0.03	N.D	N.D	N.D	N.D	N.D	N.D	N.D	N.D	N.D	N.D	N.D	0.08	N.D	N.D	N.D	N.D	0.01	0.02	0.15
S A9	N.D	N.D	N.D	N.D	N.D	N.D	N.D	N.D	0.02	0.04	<LOQ	<LOQ	N.D	0.01	N.D	N.D	N.D	N.D	N.D	N.D	N.D	0.09
S A10	N.D	0.01	0.02	N.D	N.D	N.D	N.D	N.D	N.D	N.D	N.D	N.D	N.D	0.02	<LOQ	N.D	N.D	N.D	N.D	0.01	0.01	0.08
S A11	N.D	N.D	N.D	N.D	N.D	N.D	N.D	N.D	N.D	<LOQ	N.D	N.D	N.D	0.01	N.D	N.D	N.D	N.D	N.D	N.D	N.D	0.01
S A12	N.D	N.D	N.D	N.D	N.D	N.D	N.D	N.D	N.D	N.D	N.D	<LOQ	N.D	<LOQ	N.D	N.D	N.D	N.D	N.D	N.D	N.D	–
S A13	N.D	N.D	N.D	N.D	N.D	N.D	N.D	N.D	N.D	N.D	N.D	N.D	N.D	<LOQ	N.D	N.D	N.D	N.D	N.D	N.D	N.D	-
S A14	N.D	N.D	N.D	N.D	N.D	N.D	N.D	N.D	N.D	N.D	N.D	N.D	N.D	N.D	N.D	N.D	N.D	N.D	N.D	N.D	N.D	-
S A15	N.D	N.D	N.D	N.D	N.D	N.D	N.D	N.D	N.D	N.D	N.D	N.D	N.D	N.D	N.D	N.D	N.D	N.D	N.D	N.D	N.D	-
S A16	N.D	N.D	N.D	N.D	N.D	N.D	N.D	N.D	N.D	N.D	N.D	N.D	N.D	N.D	N.D	N.D	N.D	N.D	N.D	N.D	N.D	-
S A17	N.D	N.D	N.D	N.D	N.D	N.D	N.D	N.D	N.D	N.D	N.D	N.D	N.D	N.D	N.D	N.D	N.D	N.D	N.D	N.D	<LOQ	-
S A18	N.D	N.D	N.D	N.D	N.D	N.D	N.D	N.D	N.D	N.D	N.D	N.D	N.D	N.D	N.D	N.D	N.D	N.D	N.D	N.D	N.D	-
S A19	N.D	N.D	N.D	N.D	N.D	N.D	N.D	N.D	N.D	N.D	N.D	N.D	N.D	N.D	N.D	N.D	N.D	N.D	N.D	N.D	N.D	-
S A20	N.D	N.D	N.D	N.D	N.D	N.D	N.D	N.D	N.D	N.D	N.D	N.D	N.D	N.D	N.D	N.D	N.D	N.D	N.D	N.D	N.D	-

S A21	N.D	N.D	N.D	N.D	N.D	N.D	N.D	N.D	N.D	N.D	N.D	N.D	N.D	N.D	N.D	N.D	N.D	N.D	N.D	N.D	N.D	-
S A22	N.D	N.D	N.D	N.D	N.D	N.D	N.D	N.D	N.D	N.D	N.D	N.D	N.D	<LOQ	N.D	N.D	N.D	N.D	N.D	N.D	N.D	-
S A23	N.D	N.D	N.D	N.D	N.D	N.D	N.D	N.D	N.D	N.D	N.D	N.D	N.D	N.D	N.D	N.D	N.D	N.D	N.D	N.D	N.D	-
S A24	N.D	N.D	<LOQ	N.D	N.D	N.D	N.D	N.D	N.D	<LOQ	N.D	N.D	N.D	<LOQ	N.D	N.D	N.D	N.D	N.D	N.D	<LOQ	-
S A25	N.D	N.D	<LOQ	N.D	N.D	N.D	N.D	N.D	N.D	N.D	N.D	N.D	N.D	N.D	N.D	N.D	N.D	N.D	N.D	<LOQ	N.D	-

Table S3. Lavender tea

	1	2	3	4	5	6	7	8	9	10	11	12	13	14	15	16	17	18	19	20	21	TOTAL (mg/kg)
SA1	<LOQ	0.01	0.08	N.D	N.D	N.D	N.D	N.D	N.D	N.D	N.D	N.D	N.D	<LOQ	0.09	N.D	N.D	N.D	N.D	0.02	0.02	0.22
SA2	N.D	N.D	N.D	N.D	N.D	N.D	N.D	N.D	N.D	N.D	N.D	N.D	N.D	0.20	N.D	N.D	N.D	N.D	N.D	N.D	<LOQ	0.20
SA3	N.D	N.D	N.D	N.D	N.D	N.D	N.D	N.D	0.03	N.D	0.04	<LOQ	N.D	0.03	N.D	N.D	N.D	N.D	N.D	N.D	N.D	0.09
SA4	<LOQ	<LOQ	<LOQ	N.D	N.D	N.D	N.D	N.D	N.D	N.D	N.D	N.D	N.D	0.03	57.97	N.D	N.D	N.D	N.D	<LOQ	<LOQ	0.09
SA5	N.D	N.D	N.D	N.D	N.D	N.D	N.D	N.D	N.D	N.D	N.D	N.D	N.D	0.02	N.D	N.D	N.D	N.D	N.D	N.D	<LOQ	0.02
SA6	N.D	N.D	N.D	N.D	N.D	N.D	N.D	N.D	N.D	N.D	N.D	N.D	N.D	0.02	N.D	N.D	N.D	N.D	N.D	N.D	N.D	0.02
SA7	0.00	N.D	N.D	N.D	N.D	N.D	N.D	N.D	N.D	N.D	N.D	N.D	N.D	N.D	N.D	N.D	N.D	N.D	N.D	N.D	N.D	–
SA8	0.00	N.D	N.D	N.D	N.D	N.D	N.D	N.D	N.D	N.D	N.D	N.D	<LOQ	<LOQ	N.D	N.D	N.D	N.D	N.D	N.D	N.D	-
SA9	N.D	N.D	N.D	N.D	N.D	N.D	N.D	N.D	N.D	N.D	N.D	N.D	N.D	N.D	N.D	N.D	N.D	N.D	N.D	N.D	N.D	-
SA10	N.D	N.D	N.D	N.D	N.D	N.D	N.D	N.D	N.D	N.D	N.D	N.D	N.D	N.D	N.D	N.D	N.D	N.D	N.D	N.D	N.D	-
SA11	N.D	N.D	N.D	N.D	N.D	N.D	N.D	N.D	N.D	N.D	N.D	N.D	N.D	<LOQ	N.D	N.D	N.D	N.D	N.D	N.D	N.D	-
SA12	N.D	N.D	N.D	N.D	N.D	N.D	N.D	N.D	N.D	N.D	N.D	N.D	N.D	N.D	N.D	N.D	N.D	N.D	N.D	N.D	<LOQ	-
SA13	N.D	N.D	N.D	N.D	N.D	N.D	N.D	N.D	N.D	N.D	N.D	N.D	N.D	<LOQ	N.D	N.D	N.D	N.D	N.D	N.D	N.D	-
SA14	N.D	N.D	N.D	N.D	N.D	N.D	N.D	N.D	N.D	N.D	N.D	N.D	N.D	<LOQ	N.D	N.D	N.D	N.D	N.D	N.D	N.D	-
SA15	N.D	N.D	N.D	N.D	N.D	N.D	N.D	N.D	N.D	N.D	N.D	N.D	N.D	<LOQ	<LOQ	N.D	N.D	N.D	N.D	N.D	<LOQ	-
SA16	N.D	N.D	N.D	N.D	N.D	N.D	N.D	N.D	N.D	N.D	N.D	N.D	<LOQ	N.D	<LOQ	N.D	N.D	N.D	N.D	N.D	N.D	-
SA17	N.D	N.D	N.D	N.D	N.D	N.D	N.D	N.D	N.D	N.D	N.D	N.D	<LOQ	<LOQ	N.D	N.D	N.D	N.D	N.D	N.D	N.D	-
SA18	N.D	N.D	N.D	N.D	N.D	N.D	N.D	N.D	N.D	N.D	N.D	N.D	N.D	<LOQ	N.D	N.D	N.D	N.D	N.D	N.D	N.D	-
SA19	N.D	N.D	N.D	N.D	N.D	N.D	N.D	N.D	N.D	N.D	N.D	N.D	N.D	<LOQ	N.D	N.D	N.D	N.D	N.D	N.D	N.D	-
SA20	N.D	N.D	N.D	N.D	N.D	N.D	N.D	N.D	N.D	N.D	N.D	N.D	N.D	N.D	N.D	N.D	N.D	N.D	N.D	N.D	N.D	-

Table S4. Chamomile tea

	1	2	3	4	5	6	7	8	9	10	11	12	13	14	15	16	17	18	19	20	21	Total (mg/kg)
S A1	0.08	N.D	N.D	<LOQ	N.D	N.D	N.D	N.D	N.D	N.D	N.D	N.D	N.D	0.03	N.D	0.02	N.D	N.D	N.D	N.D	N.D	0.12
S A2	N.D	N.D	N.D	13.05	N.D	N.D	N.D	N.D	N.D	<LOQ	N.D	N.D	N.D	0.03	N.D	0.07	N.D	N.D	N.D	N.D	<LOQ	0.11
S A3	N.D	N.D	N.D	<LOQ	N.D	N.D	N.D	N.D	<LOQ	0.03	<LOQ	<LOQ	<LOQ	0.03	N.D	N.D	N.D	N.D	N.D	N.D	N.D	0.06
S A4	N.D	N.D	N.D	N.D	N.D	N.D	N.D	N.D	<LOQ	0.06	N.D	N.D	<LOQ	N.D	N.D	N.D	N.D	N.D	N.D	N.D	N.D	0.06
S A5	0.03	N.D	N.D	N.D	N.D	N.D	N.D	N.D	N.D	N.D	N.D	N.D	<LOQ	0.03	N.D	<LOQ	N.D	N.D	N.D	N.D	N.D	0.06
S A6	N.D	N.D	N.D	N.D	N.D	N.D	N.D	N.D	N.D	0.03	N.D	<LOQ	<LOQ	0.01	N.D	N.D	N.D	N.D	N.D	N.D	N.D	0.04
S A7	N.D	N.D	N.D	N.D	N.D	N.D	N.D	N.D	N.D	N.D	N.D	N.D	<LOQ	0.03	N.D	N.D	N.D	N.D	N.D	N.D	N.D	0.03
S A8	N.D	N.D	N.D	N.D	N.D	N.D	N.D	N.D	N.D	N.D	N.D	N.D	N.D	0.02	N.D	N.D	N.D	N.D	N.D	N.D	N.D	0.02
S A9	N.D	N.D	N.D	N.D	N.D	N.D	N.D	N.D	N.D	N.D	N.D	N.D	N.D	N.D	N.D	N.D	N.D	N.D	N.D	N.D	N.D	-
S A10	N.D	N.D	N.D	N.D	N.D	N.D	N.D	N.D	N.D	N.D	N.D	N.D	N.D	<LOQ	N.D	N.D	N.D	N.D	N.D	N.D	N.D	-
S A11	N.D	N.D	N.D	N.D	N.D	N.D	N.D	N.D	N.D	N.D	N.D	N.D	N.D	N.D	N.D	N.D	N.D	N.D	N.D	N.D	N.D	-
S A12	N.D	N.D	N.D	N.D	N.D	N.D	N.D	N.D	N.D	N.D	N.D	N.D	N.D	<LOQ	N.D	N.D	N.D	N.D	N.D	N.D	N.D	-
S A13	N.D	N.D	N.D	N.D	N.D	N.D	N.D	N.D	N.D	N.D	N.D	N.D	N.D	N.D	N.D	N.D	N.D	N.D	N.D	N.D	N.D	-
S A14	N.D	N.D	N.D	N.D	N.D	N.D	N.D	N.D	N.D	N.D	N.D	N.D	N.D	<LOQ	N.D	N.D	N.D	N.D	N.D	N.D	N.D	-
S A15	N.D	N.D	N.D	N.D	N.D	N.D	N.D	N.D	N.D	N.D	N.D	N.D	N.D	N.D	N.D	N.D	N.D	N.D	N.D	N.D	N.D	-
S A16	N.D	N.D	N.D	N.D	N.D	N.D	N.D	N.D	N.D	N.D	N.D	<LOQ	N.D	<LOQ	N.D	N.D	N.D	N.D	N.D	<LOQ	N.D	-
S A17	N.D	N.D	N.D	N.D	N.D	N.D	N.D	N.D	N.D	N.D	N.D	N.D	N.D	N.D	N.D	N.D	N.D	N.D	N.D	N.D	N.D	-
S A18	N.D	N.D	N.D	N.D	N.D	N.D	N.D	N.D	N.D	N.D	N.D	N.D	N.D	N.D	N.D	N.D	N.D	N.D	N.D	N.D	N.D	-
S A19	N.D	N.D	N.D	N.D	N.D	N.D	N.D	N.D	N.D	<LOQ	N.D	N.D	<LOQ	<LOQ	N.D	<LOQ	N.D	N.D	N.D	N.D	N.D	-
S A20	N.D	N.D	N.D	N.D	N.D	N.D	N.D	N.D	N.D	N.D	N.D	N.D	N.D	N.D	N.D	N.D	N.D	N.D	N.D	N.D	N.D	-
S A21	N.D	N.D	N.D	N.D	N.D	N.D	N.D	N.D	N.D	N.D	N.D	N.D	N.D	N.D	N.D	N.D	N.D	N.D	N.D	N.D	N.D	-

Table S5. Lemon balm

	1	2	3	4	5	6	7	8	9	10	11	12	13	14	15	16	17	18	19	20	21	T O T A L (mg/kg)
S A1	0.00	0.10	0.40	<LOQ	N.D	<LOQ	N.D	N.D	N.D	N.D	N.D	<LOQ	<LOQ	<LOQ	0.74	<LOQ	N.D	N.D	N.D	0.35	0.29	1.88
S A2	N.D	0.03	0.20	N.D	N.D	N.D	N.D	N.D	N.D	N.D	N.D	N.D	N.D	N.D	0.28	N.D	N.D	N.D	N.D	0.15	0.07	0.74
S A3	N.D	<LOQ	0.03	N.D	N.D	N.D	N.D	N.D	N.D	N.D	N.D	N.D	N.D	N.D	0.12	N.D	N.D	N.D	N.D	0.03	0.01	0.18
S A4	N.D	N.D	<LOQ	N.D	N.D	N.D	<LOQ	N.D	N.D	N.D	0.10	N.D	N.D	<LOQ	N.D	N.D	N.D	N.D	N.D	N.D	<LOQ	0.10
S A5	N.D	N.D	<LOQ	N.D	N.D	N.D	N.D	N.D	N.D	N.D	N.D	N.D	N.D	N.D	0.08	N.D	N.D	N.D	N.D	<LOQ	<LOQ	0.08
S A6	N.D	N.D	N.D	N.D	N.D	N.D	N.D	N.D	N.D	N.D	N.D	N.D	N.D	0.06	N.D	N.D	N.D	N.D	N.D	N.D	N.D	0.06
S A7	N.D	<LOQ	N.D	N.D	N.D	N.D	N.D	N.D	N.D	N.D	N.D	N.D	N.D	N.D	N.D	N.D	N.D	N.D	N.D	N.D	<LOQ	-
S A8	N.D	N.D	N.D	N.D	N.D	N.D	N.D	N.D	N.D	N.D	N.D	N.D	<LOQ	N.D	N.D	N.D	N.D	N.D	N.D	N.D	N.D	-
S A9	N.D	<LOQ	N.D	N.D	N.D	N.D	N.D	N.D	N.D	N.D	N.D	N.D	N.D	N.D	N.D	N.D	N.D	N.D	N.D	<LOQ	<LOQ	-
S A10	N.D	N.D	N.D	N.D	N.D	N.D	N.D	N.D	N.D	N.D	N.D	N.D	N.D	N.D	N.D	N.D	N.D	N.D	N.D	N.D	N.D	-
S A11	N.D	N.D	<LOQ	N.D	N.D	N.D	N.D	N.D	N.D	N.D	N.D	N.D	N.D	N.D	N.D	N.D	N.D	N.D	N.D	N.D	<LOQ	-
S A12	N.D	N.D	N.D	N.D	N.D	N.D	N.D	N.D	N.D	N.D	N.D	N.D	N.D	N.D	N.D	N.D	N.D	N.D	N.D	N.D	<LOQ	-
S A13	N.D	N.D	N.D	N.D	N.D	N.D	N.D	N.D	N.D	N.D	N.D	N.D	N.D	N.D	N.D	N.D	N.D	N.D	N.D	N.D	N.D	-
S A14	N.D	N.D	N.D	N.D	N.D	N.D	N.D	N.D	N.D	N.D	N.D	N.D	N.D	N.D	N.D	N.D	N.D	N.D	N.D	N.D	N.D	-
S A15	<LOQ	N.D	N.D	N.D	N.D	N.D	N.D	N.D	N.D	N.D	N.D	N.D	N.D	<LOQ	N.D	N.D	N.D	N.D	N.D	<LOQ	<LOQ	-
S A16	N.D	N.D	N.D	N.D	N.D	N.D	N.D	N.D	N.D	N.D	N.D	N.D	N.D	N.D	N.D	N.D	N.D	N.D	N.D	N.D	<LOQ	-
S A17	N.D	<LOQ	N.D	N.D	N.D	N.D	N.D	N.D	N.D	N.D	N.D	N.D	N.D	N.D	N.D	<LOQ	N.D	N.D	N.D	N.D	<LOQ	-
S A18	N.D	N.D	N.D	N.D	N.D	N.D	N.D	N.D	N.D	N.D	N.D	N.D	N.D	N.D	N.D	N.D	N.D	N.D	N.D	N.D	N.D	-

Table S6. Mixed tea

	1	2	3	4	5	6	7	8	9	10	11	12	13	14	15	16	17	18	19	20	21	Total (mg/kg)
SA1	0.16	N.D	N.D	<LOQ	N.D	N.D	0.18	N.D	0.34	0.16	0.53	0.12	N.D	<LOQ	N.D	<LOQ	N.D	N.D	N.D	N.D	N.D	1.49
SA2	<LOQ	0.11	0.19	N.D	N.D	N.D	N.D	N.D	N.D	<LOQ	<LOQ	<LOQ	N.D	N.D	0.07	N.D	N.D	N.D	N.D	0.02	0.06	0.46
SA3	N.D	<LOQ	0.06	N.D	N.D	N.D	N.D	N.D	N.D	<LOQ	N.D	N.D	<LOQ	0.04	0.09	N.D	N.D	N.D	N.D	0.03	<LOQ	0.21
SA4	N.D	0.03	<LOQ	N.D	N.D	N.D	N.D	N.D	N.D	N.D	N.D	N.D	<LOQ	N.D	0.11	N.D	N.D	N.D	N.D	<LOQ	0.03	0.17
SA5	<LOQ	N.D	N.D	N.D	N.D	N.D	N.D	N.D	0.11	0.04	N.D	N.D	<LOQ	N.D	N.D	N.D	N.D	N.D	N.D	N.D	N.D	0.15
SA6	N.D	N.D	N.D	N.D	N.D	N.D	N.D	N.D	0.04	0.03	N.D	N.D	<LOQ	N.D	N.D	N.D	N.D	N.D	N.D	N.D	N.D	0.07
SA7	N.D	0.01	N.D	N.D	N.D	N.D	N.D	N.D	N.D	N.D	N.D	N.D	N.D	N.D	N.D	N.D	N.D	N.D	N.D	N.D	<LOQ	0.01
SA8	N.D	N.D	N.D	N.D	N.D	N.D	N.D	N.D	N.D	N.D	N.D	N.D	N.D	N.D	N.D	N.D	N.D	N.D	N.D	N.D	<LOQ	-
SA9	N.D	N.D	N.D	N.D	N.D	N.D	N.D	N.D	N.D	N.D	N.D	N.D	N.D	N.D	N.D	N.D	N.D	N.D	N.D	N.D	N.D	-
SA10	<LOQ	N.D	N.D	N.D	N.D	N.D	N.D	N.D	N.D	N.D	N.D	N.D	N.D	N.D	N.D	N.D	N.D	N.D	N.D	N.D	N.D	-
SA11	N.D	N.D	N.D	N.D	N.D	N.D	N.D	N.D	N.D	N.D	N.D	N.D	N.D	N.D	N.D	N.D	N.D	N.D	N.D	N.D	<LOQ	-
SA12	N.D	N.D	N.D	N.D	N.D	N.D	N.D	N.D	N.D	N.D	N.D	N.D	N.D	N.D	N.D	N.D	N.D	N.D	N.D	N.D	N.D	-
SA13	N.D	N.D	N.D	N.D	N.D	N.D	N.D	N.D	N.D	N.D	N.D	N.D	N.D	N.D	N.D	N.D	N.D	N.D	N.D	N.D	N.D	-
SA14	N.D	N.D	N.D	N.D	N.D	N.D	N.D	N.D	N.D	N.D	N.D	N.D	N.D	N.D	N.D	N.D	N.D	N.D	N.D	N.D	N.D	-
SA15	N.D	N.D	N.D	N.D	N.D	N.D	N.D	N.D	N.D	N.D	N.D	N.D	N.D	N.D	N.D	N.D	N.D	N.D	N.D	N.D	N.D	-
SA16	N.D	N.D	N.D	N.D	N.D	N.D	N.D	N.D	N.D	N.D	N.D	N.D	N.D	N.D	N.D	N.D	N.D	N.D	N.D	N.D	N.D	-
SA17	N.D	N.D	N.D	N.D	N.D	N.D	N.D	N.D	N.D	N.D	N.D	N.D	<LOQ	N.D	N.D	N.D	N.D	N.D	N.D	N.D	N.D	-
SA18	N.D	N.D	N.D	N.D	N.D	N.D	N.D	N.D	N.D	N.D	N.D	N.D	N.D	N.D	N.D	N.D	N.D	N.D	N.D	N.D	N.D	-
SA19	N.D	N.D	N.D	N.D	N.D	N.D	N.D	N.D	N.D	N.D	N.D	N.D	N.D	N.D	N.D	N.D	N.D	N.D	N.D	N.D	N.D	-
SA20	N.D	N.D	N.D	N.D	N.D	N.D	N.D	N.D	N.D	N.D	N.D	N.D	N.D	N.D	N.D	N.D	N.D	N.D	N.D	N.D	N.D	-
SA21	N.D	N.D	N.D	N.D	N.D	N.D	N.D	N.D	N.D	N.D	N.D	N.D	N.D	N.D	N.D	N.D	N.D	N.D	N.D	N.D	N.D	-
SA22	<LOQ	N.D	N.D	N.D	N.D	N.D	N.D	N.D	N.D	N.D	N.D	N.D	N.D	N.D	N.D	N.D	N.D	N.D	N.D	N.D	N.D	-
SA23	N.D	N.D	N.D	N.D	N.D	N.D	N.D	N.D	N.D	N.D	N.D	N.D	N.D	N.D	N.D	N.D	N.D	N.D	N.D	N.D	N.D	-
SA24	<LOQ	N.D	N.D	N.D	N.D	N.D	N.D	N.D	N.D	N.D	N.D	N.D	N.D	N.D	N.D	<LOQ	N.D	N.D	N.D	N.D	N.D	-
SA25	N.D	N.D	N.D	N.D	N.D	N.D	N.D	N.D	N.D	N.D	N.D	N.D	N.D	N.D	N.D	N.D	N.D	N.D	N.D	N.D	N.D	-

Table S7. Black tea

	1	2	3	4	5	6	7	8	9	10	11	12	13	14	15	16	17	18	19	20	21	TOTAL(mg/kg)
SA1	N.D	N.D	N.D	N.D	N.D	N.D	0.13	N.D	0.03	N.D	N.D	N.D	<LOQ	N.D	N.D	N.D	N.D	N.D	N.D	N.D	N.D	0.16
SA2	N.D	N.D	N.D	N.D	N.D	N.D	0.05	N.D	0.07	N.D	N.D	N.D	N.D	N.D	N.D	N.D	N.D	N.D	N.D	N.D	N.D	0.12
SA3	N.D	N.D	N.D	N.D	N.D	N.D	N.D	N.D	0.01	N.D	0.01	N.D	N.D	0.05	N.D	N.D	N.D	N.D	N.D	N.D	N.D	0.07
SA4	N.D	N.D	N.D	N.D	N.D	N.D	N.D	N.D	N.D	N.D	N.D	N.D	N.D	<LOQ	N.D	N.D	N.D	N.D	N.D	N.D	N.D	-
SA5	N.D	N.D	N.D	N.D	N.D	N.D	N.D	N.D	N.D	N.D	N.D	N.D	N.D	N.D	N.D	N.D	N.D	N.D	N.D	N.D	<LOQ	-
SA6	N.D	N.D	N.D	N.D	N.D	N.D	N.D	N.D	N.D	N.D	N.D	N.D	N.D	N.D	N.D	N.D	N.D	N.D	N.D	N.D	N.D	-
SA7	N.D	N.D	N.D	N.D	N.D	N.D	N.D	N.D	N.D	N.D	N.D	N.D	N.D	N.D	N.D	N.D	N.D	N.D	N.D	N.D	N.D	-
SA8	N.D	N.D	N.D	N.D	N.D	N.D	N.D	N.D	N.D	N.D	N.D	N.D	N.D	N.D	N.D	N.D	N.D	N.D	N.D	N.D	N.D	-
SA9	N.D	N.D	N.D	N.D	N.D	N.D	N.D	N.D	N.D	N.D	N.D	N.D	N.D	N.D	N.D	N.D	N.D	N.D	N.D	N.D	N.D	-
SA10	N.D	N.D	N.D	N.D	N.D	N.D	N.D	N.D	N.D	N.D	N.D	N.D	N.D	N.D	N.D	N.D	N.D	N.D	N.D	N.D	N.D	-
SA11	N.D	N.D	N.D	N.D	N.D	N.D	N.D	N.D	N.D	N.D	N.D	N.D	N.D	N.D	N.D	N.D	N.D	N.D	N.D	N.D	N.D	-
SA12	N.D	N.D	N.D	N.D	N.D	N.D	N.D	N.D	N.D	N.D	N.D	N.D	N.D	N.D	N.D	N.D	N.D	N.D	N.D	N.D	N.D	-
SA13	N.D	N.D	N.D	N.D	N.D	N.D	N.D	N.D	N.D	N.D	N.D	N.D	N.D	N.D	N.D	N.D	N.D	N.D	N.D	N.D	N.D	-
SA14	N.D	N.D	N.D	N.D	N.D	N.D	N.D	N.D	N.D	N.D	N.D	N.D	N.D	N.D	N.D	N.D	N.D	N.D	N.D	N.D	N.D	-
SA15	N.D	N.D	N.D	N.D	N.D	N.D	N.D	N.D	N.D	N.D	N.D	N.D	N.D	<LOQ	N.D	N.D	N.D	N.D	N.D	N.D	N.D	-
SA16	N.D	N.D	N.D	N.D	N.D	N.D	N.D	N.D	N.D	N.D	N.D	N.D	N.D	N.D	N.D	<LOQ	N.D	N.D	N.D	N.D	N.D	-
SA17	N.D	N.D	N.D	N.D	N.D	N.D	N.D	N.D	N.D	N.D	N.D	N.D	N.D	N.D	N.D	N.D	N.D	N.D	N.D	N.D	N.D	-
SA18	N.D	N.D	N.D	N.D	N.D	N.D	N.D	N.D	N.D	N.D	N.D	N.D	N.D	N.D	N.D	N.D	N.D	N.D	N.D	N.D	N.D	-
SA19	N.D	N.D	N.D	N.D	N.D	N.D	<LOQ	N.D	N.D	N.D	N.D	N.D	N.D	<LOQ	N.D	N.D	N.D	N.D	N.D	N.D	N.D	-
SA20	N.D	N.D	N.D	N.D	N.D	N.D	N.D	N.D	N.D	N.D	N.D	N.D	N.D	N.D	N.D	N.D	N.D	N.D	N.D	N.D	N.D	-
SA21	N.D	N.D	N.D	N.D	N.D	N.D	N.D	N.D	N.D	N.D	N.D	N.D	N.D	N.D	N.D	N.D	N.D	N.D	N.D	N.D	N.D	-
SA22	N.D	N.D	N.D	N.D	N.D	N.D	N.D	N.D	N.D	N.D	N.D	N.D	N.D	N.D	N.D	N.D	N.D	N.D	N.D	N.D	N.D	-
SA23	N.D	N.D	N.D	N.D	N.D	N.D	N.D	N.D	N.D	N.D	N.D	N.D	N.D	N.D	N.D	N.D	N.D	N.D	N.D	N.D	N.D	-
SA24	N.D	N.D	N.D	N.D	N.D	N.D	N.D	N.D	N.D	N.D	N.D	N.D	N.D	N.D	N.D	N.D	N.D	N.D	N.D	N.D	N.D	-
SA25	N.D	N.D	N.D	N.D	N.D	N.D	N.D	N.D	N.D	N.D	N.D	N.D	N.D	N.D	N.D	N.D	N.D	N.D	N.D	N.D	N.D	-
SA26	N.D	N.D	N.D	N.D	N.D	N.D	N.D	N.D	N.D	N.D	N.D	N.D	N.D	N.D	N.D	N.D	N.D	N.D	N.D	N.D	N.D	-
SA27	N.D	N.D	N.D	N.D	N.D	N.D	N.D	N.D	N.D	N.D	N.D	N.D	N.D	N.D	N.D	N.D	N.D	N.D	N.D	N.D	N.D	-
SA28	N.D	N.D	N.D	N.D	N.D	N.D	N.D	N.D	N.D	N.D	N.D	N.D	N.D	N.D	N.D	N.D	N.D	N.D	N.D	N.D	N.D	-

S A29	N.D	N.D	N.D	N.D	N.D	N.D	N.D	N.D	N.D	N.D	N.D	N.D	N.D	N.D	N.D	N.D	N.D	N.D	N.D	N.D	N.D	-
S A30	N.D	N.D	N.D	N.D	N.D	N.D	N.D	N.D	N.D	N.D	N.D	N.D	N.D	N.D	N.D	N.D	N.D	N.D	N.D	N.D	N.D	-
S A31	N.D	N.D	N.D	N.D	N.D	N.D	N.D	N.D	N.D	N.D	N.D	N.D	N.D	N.D	N.D	N.D	N.D	N.D	N.D	N.D	N.D	-

Table S8. Maté tea

	1	2	3	4	5	6	7	8	9	10	11	12	13	14	15	16	17	18	19	20	21	T O T A L (mg/kg)
마테차1	<LOQ	0.01	0.03	N.D	N.D	<LOQ	N.D	N.D	N.D	<LOQ	N.D	<LOQ	N.D	N.D	N.D	N.D	N.D	N.D	N.D	N.D	N.D	0.04
마테차2	N.D	N.D	N.D	N.D	N.D	N.D	<LOQ	N.D	N.D	N.D	<LOQ	N.D	N.D	N.D	N.D	N.D	N.D	N.D	N.D	N.D	N.D	-
마테차3	N.D	N.D	N.D	N.D	N.D	N.D	N.D	N.D	N.D	N.D	N.D	N.D	N.D	N.D	N.D	N.D	N.D	N.D	N.D	N.D	N.D	-
마테차4	N.D	N.D	N.D	N.D	N.D	N.D	N.D	N.D	N.D	N.D	N.D	N.D	N.D	N.D	N.D	N.D	N.D	N.D	N.D	N.D	N.D	-
마테차5	N.D	<LOQ	N.D	N.D	N.D	N.D	N.D	N.D	N.D	N.D	N.D	N.D	N.D	N.D	N.D	N.D	N.D	N.D	N.D	N.D	N.D	-
마테차6	N.D	N.D	N.D	N.D	N.D	N.D	N.D	N.D	N.D	N.D	N.D	N.D	N.D	N.D	N.D	N.D	N.D	N.D	N.D	N.D	N.D	-
마테차7	N.D	N.D	N.D	N.D	N.D	N.D	N.D	N.D	N.D	N.D	N.D	N.D	N.D	N.D	N.D	N.D	N.D	N.D	N.D	N.D	N.D	-
마테차8	<LOQ	N.D	N.D	N.D	N.D	N.D	N.D	N.D	N.D	N.D	N.D	N.D	N.D	N.D	N.D	N.D	N.D	N.D	N.D	N.D	N.D	-
마테차9	N.D	N.D	N.D	N.D	N.D	N.D	N.D	N.D	N.D	N.D	N.D	N.D	N.D	N.D	N.D	<LOQ	N.D	N.D	N.D	N.D	N.D	-
마테차10	N.D	N.D	N.D	N.D	N.D	N.D	N.D	N.D	N.D	N.D	N.D	N.D	N.D	N.D	N.D	N.D	N.D	N.D	N.D	N.D	N.D	-
마테차11	N.D	N.D	N.D	N.D	N.D	N.D	N.D	N.D	N.D	N.D	N.D	N.D	N.D	N.D	N.D	N.D	N.D	N.D	N.D	N.D	N.D	-
마테차12	N.D	N.D	N.D	N.D	N.D	N.D	N.D	N.D	N.D	N.D	N.D	N.D	N.D	N.D	N.D	N.D	N.D	N.D	N.D	N.D	N.D	-
마테차13	N.D	N.D	N.D	N.D	N.D	N.D	N.D	N.D	N.D	N.D	N.D	N.D	N.D	N.D	N.D	N.D	N.D	N.D	N.D	N.D	N.D	-
마테차14	N.D	N.D	N.D	N.D	N.D	N.D	N.D	N.D	N.D	N.D	N.D	N.D	N.D	N.D	N.D	N.D	N.D	N.D	N.D	N.D	N.D	-
마테차15	N.D	N.D	N.D	N.D	N.D	N.D	N.D	N.D	N.D	N.D	N.D	N.D	N.D	N.D	N.D	<LOQ	N.D	N.D	N.D	N.D	N.D	-
마테차16	N.D	N.D	N.D	N.D	N.D	N.D	N.D	N.D	N.D	N.D	N.D	N.D	N.D	N.D	N.D	N.D	N.D	N.D	N.D	N.D	N.D	-

Table S9. Green tea

	1	2	3	4	5	6	7	8	9	10	11	12	13	14	15	16	17	18	19	20	21	TOTAL (mg/kg)
SA1	N.D	N.D	N.D	N.D	N.D	N.D	N.D	N.D	N.D	N.D	N.D	N.D	N.D	N.D	N.D	N.D	N.D	N.D	N.D	N.D	N.D	0.00
SA2	N.D	N.D	N.D	N.D	N.D	N.D	N.D	N.D	N.D	N.D	N.D	N.D	<LOQ	N.D	N.D	N.D	N.D	N.D	N.D	N.D	N.D	0.00
SA3	N.D	N.D	N.D	N.D	N.D	N.D	N.D	N.D	N.D	N.D	N.D	N.D	N.D	N.D	N.D	N.D	N.D	N.D	N.D	N.D	N.D	0.00
SA4	N.D	N.D	N.D	N.D	N.D	N.D	N.D	N.D	N.D	N.D	N.D	N.D	<LOQ	N.D	N.D	N.D	N.D	N.D	N.D	N.D	N.D	0.00
SA5	N.D	N.D	N.D	N.D	N.D	N.D	N.D	N.D	N.D	N.D	N.D	N.D	<LOQ	N.D	N.D	N.D	N.D	N.D	N.D	N.D	N.D	0.00
SA6	N.D	N.D	N.D	N.D	N.D	N.D	N.D	N.D	N.D	N.D	N.D	N.D	N.D	N.D	N.D	N.D	N.D	N.D	N.D	N.D	N.D	0.00
SA7	N.D	N.D	N.D	N.D	N.D	N.D	N.D	N.D	N.D	N.D	N.D	N.D	N.D	N.D	N.D	N.D	N.D	N.D	N.D	N.D	N.D	0.00
SA8	N.D	N.D	N.D	N.D	N.D	N.D	N.D	N.D	N.D	N.D	N.D	N.D	<LOQ	N.D	N.D	N.D	N.D	N.D	N.D	N.D	N.D	0.00
SA9	N.D	N.D	N.D	N.D	N.D	N.D	N.D	N.D	N.D	N.D	N.D	N.D	N.D	N.D	N.D	N.D	N.D	N.D	N.D	N.D	N.D	0.00
SA10	N.D	N.D	N.D	N.D	N.D	N.D	N.D	N.D	N.D	N.D	N.D	N.D	N.D	N.D	N.D	N.D	N.D	N.D	N.D	N.D	N.D	0.00
SA11	N.D	N.D	N.D	N.D	N.D	N.D	N.D	N.D	N.D	N.D	N.D	N.D	<LOQ	N.D	N.D	N.D	N.D	N.D	N.D	N.D	N.D	0.00
SA12	N.D	N.D	N.D	N.D	N.D	N.D	N.D	N.D	N.D	N.D	N.D	N.D	N.D	N.D	N.D	N.D	N.D	N.D	N.D	N.D	N.D	0.00
SA13	N.D	N.D	N.D	N.D	N.D	N.D	N.D	N.D	N.D	N.D	N.D	N.D	N.D	N.D	N.D	N.D	N.D	N.D	N.D	N.D	N.D	0.00
SA14	N.D	N.D	N.D	N.D	N.D	N.D	N.D	N.D	N.D	N.D	N.D	N.D	N.D	N.D	N.D	N.D	N.D	N.D	N.D	N.D	N.D	0.00
SA15	N.D	N.D	N.D	N.D	N.D	N.D	N.D	N.D	N.D	N.D	N.D	N.D	N.D	N.D	N.D	N.D	N.D	N.D	N.D	N.D	N.D	0.00
SA16	N.D	N.D	N.D	N.D	N.D	N.D	N.D	N.D	N.D	N.D	N.D	N.D	<LOQ	N.D	N.D	N.D	N.D	N.D	N.D	N.D	N.D	0.00
SA17	N.D	N.D	N.D	N.D	N.D	N.D	N.D	N.D	N.D	N.D	N.D	N.D	N.D	N.D	N.D	N.D	N.D	N.D	N.D	N.D	N.D	0.00
SA18	N.D	N.D	N.D	N.D	N.D	N.D	N.D	N.D	N.D	N.D	N.D	N.D	N.D	N.D	N.D	N.D	N.D	N.D	N.D	N.D	N.D	0.00
SA19	N.D	N.D	N.D	N.D	N.D	N.D	N.D	N.D	N.D	N.D	N.D	N.D	N.D	N.D	N.D	N.D	N.D	N.D	N.D	N.D	N.D	0.00

S A20	N.D	N.D	N.D	N.D	N.D	N.D	N.D	N.D	N.D	N.D	N.D	N.D	N.D	N.D	N.D	N.D	N.D	N.D	N.D	N.D	N.D	0.00
S A21	N.D	N.D	N.D	N.D	N.D	N.D	N.D	N.D	N.D	N.D	N.D	N.D	N.D	N.D	N.D	N.D	N.D	N.D	N.D	N.D	N.D	0.00
S A22	N.D	N.D	N.D	N.D	N.D	N.D	N.D	N.D	N.D	N.D	N.D	N.D	N.D	N.D	N.D	N.D	N.D	N.D	N.D	N.D	N.D	0.00
S A23	N.D	N.D	N.D	N.D	N.D	N.D	N.D	N.D	N.D	N.D	N.D	N.D	N.D	N.D	N.D	N.D	N.D	N.D	N.D	N.D	N.D	0.00
S A24	N.D	N.D	N.D	N.D	N.D	N.D	N.D	N.D	N.D	N.D	N.D	N.D	N.D	N.D	N.D	N.D	N.D	N.D	N.D	N.D	N.D	0.00
S A25	N.D	N.D	N.D	N.D	N.D	N.D	N.D	N.D	N.D	N.D	N.D	N.D	N.D	N.D	N.D	N.D	N.D	N.D	N.D	N.D	N.D	0.00
S A26	N.D	N.D	N.D	N.D	N.D	N.D	N.D	N.D	N.D	N.D	N.D	N.D	N.D	N.D	N.D	N.D	N.D	N.D	N.D	N.D	N.D	0.00
S A27	N.D	N.D	N.D	N.D	N.D	N.D	N.D	N.D	N.D	N.D	N.D	N.D	N.D	N.D	N.D	N.D	N.D	N.D	N.D	N.D	N.D	0.00
S A28	N.D	N.D	N.D	N.D	N.D	N.D	N.D	N.D	N.D	N.D	N.D	N.D	N.D	N.D	N.D	N.D	N.D	N.D	N.D	N.D	N.D	0.00
S A29	N.D	N.D	N.D	N.D	N.D	N.D	N.D	N.D	N.D	N.D	N.D	N.D	N.D	N.D	N.D	<LOQ	N.D	N.D	N.D	N.D	N.D	0.00
S A30	N.D	N.D	N.D	N.D	N.D	N.D	N.D	N.D	N.D	N.D	N.D	N.D	N.D	N.D	N.D	N.D	N.D	N.D	N.D	N.D	N.D	0.00
S A31	N.D	N.D	N.D	N.D	N.D	N.D	N.D	N.D	N.D	N.D	N.D	N.D	N.D	N.D	N.D	N.D	N.D	N.D	N.D	N.D	N.D	0.00
S A32	N.D	N.D	N.D	N.D	N.D	N.D	N.D	N.D	N.D	N.D	N.D	N.D	N.D	N.D	N.D	N.D	N.D	N.D	N.D	N.D	N.D	0.00

Table S10. Oolong tea

	1	2	3	4	5	6	7	8	9	10	11	12	13	14	15	16	17	18	19	20	21	TOTAL (mg/kg)
S A1	N.D	N.D	N.D	N.D	N.D	N.D	N.D	N.D	N.D	N.D	N.D	N.D	N.D	N.D	N.D	N.D	N.D	N.D	N.D	N.D	N.D	0.00
S A2	N.D	N.D	N.D	N.D	N.D	N.D	N.D	N.D	N.D	N.D	N.D	<LOQ	<LOQ	N.D	N.D	N.D	N.D	N.D	N.D	N.D	N.D	0.00
S A3	N.D	N.D	N.D	N.D	N.D	N.D	N.D	N.D	N.D	N.D	N.D	N.D	<LOQ	<LOQ	N.D	<LOQ	N.D	N.D	N.D	N.D	N.D	0.00
S A4	N.D	N.D	N.D	N.D	N.D	N.D	N.D	N.D	N.D	N.D	N.D	N.D	<LOQ	N.D	N.D	N.D	N.D	N.D	N.D	N.D	N.D	0.00
S A5	N.D	N.D	N.D	N.D	N.D	N.D	N.D	N.D	N.D	N.D	N.D	N.D	N.D	N.D	N.D	N.D	N.D	N.D	N.D	N.D	N.D	0.00
S A6	N.D	N.D	N.D	N.D	N.D	N.D	N.D	N.D	N.D	N.D	N.D	N.D	N.D	N.D	N.D	N.D	N.D	N.D	N.D	N.D	N.D	0.00

S A7	N.D	N.D	N.D	N.D	N.D	N.D	N.D	N.D	N.D	N.D	N.D	N.D	N.D	N.D	N.D	N.D	N.D	N.D	N.D	N.D	N.D	0.00
S A8	N.D	N.D	N.D	N.D	N.D	N.D	N.D	N.D	N.D	N.D	N.D	N.D	N.D	N.D	N.D	N.D	N.D	N.D	N.D	N.D	N.D	0.00
S A9	N.D	N.D	N.D	N.D	N.D	N.D	N.D	N.D	N.D	N.D	N.D	N.D	N.D	N.D	N.D	N.D	N.D	N.D	N.D	N.D	N.D	0.00
S A10	N.D	N.D	N.D	N.D	N.D	N.D	N.D	N.D	N.D	N.D	N.D	N.D	N.D	N.D	N.D	N.D	N.D	N.D	N.D	N.D	N.D	0.00
S A11	N.D	N.D	N.D	N.D	N.D	N.D	N.D	N.D	N.D	N.D	N.D	N.D	N.D	N.D	N.D	N.D	N.D	N.D	N.D	N.D	N.D	0.00
S A12	N.D	N.D	N.D	N.D	N.D	N.D	N.D	N.D	N.D	N.D	N.D	N.D	N.D	N.D	N.D	N.D	N.D	N.D	N.D	N.D	N.D	0.00
S A13	N.D	N.D	N.D	N.D	N.D	N.D	N.D	N.D	N.D	N.D	N.D	N.D	N.D	N.D	N.D	N.D	N.D	N.D	N.D	N.D	N.D	0.00
S A14	N.D	N.D	N.D	N.D	N.D	N.D	N.D	N.D	N.D	N.D	N.D	N.D	N.D	N.D	N.D	N.D	N.D	N.D	N.D	N.D	N.D	0.00
S A15	N.D	N.D	N.D	N.D	N.D	N.D	N.D	N.D	N.D	N.D	N.D	N.D	N.D	N.D	N.D	N.D	N.D	N.D	N.D	N.D	N.D	0.00
S A16	N.D	N.D	N.D	N.D	N.D	N.D	N.D	N.D	N.D	N.D	N.D	N.D	<LOQ	N.D	N.D	N.D	N.D	N.D	N.D	N.D	N.D	0.00
S A17	N.D	N.D	N.D	N.D	N.D	N.D	N.D	N.D	N.D	N.D	N.D	N.D	N.D	N.D	N.D	N.D	N.D	N.D	N.D	N.D	N.D	0.00
S A18	N.D	N.D	N.D	N.D	N.D	N.D	N.D	N.D	N.D	N.D	N.D	N.D	N.D	N.D	N.D	N.D	N.D	N.D	N.D	N.D	N.D	0.00
S A19	N.D	N.D	N.D	N.D	N.D	N.D	N.D	N.D	N.D	N.D	N.D	N.D	N.D	N.D	N.D	N.D	N.D	N.D	N.D	N.D	N.D	0.00
S A20	N.D	N.D	N.D	N.D	N.D	N.D	N.D	N.D	N.D	N.D	N.D	N.D	<LOQ	N.D	N.D	N.D	N.D	N.D	N.D	N.D	N.D	0.00
S A21	N.D	N.D	N.D	N.D	N.D	N.D	N.D	N.D	N.D	N.D	N.D	N.D	N.D	N.D	N.D	N.D	N.D	N.D	N.D	N.D	N.D	0.00

Table S11. Chrysanthemum tea

	1	2	3	4	5	6	7	8	9	10	11	12	13	14	15	16	17	18	19	20	21	T O T A L (mg/kg)
S A1	N.D	N.D	N.D	N.D	N.D	N.D	N.D	N.D	N.D	N.D	N.D	N.D	N.D	N.D	N.D	N.D	N.D	N.D	N.D	N.D	N.D	0.00
S A2	N.D	N.D	N.D	N.D	N.D	N.D	N.D	N.D	N.D	N.D	N.D	N.D	N.D	N.D	N.D	N.D	N.D	N.D	N.D	N.D	N.D	0.00
S A3	N.D	N.D	N.D	N.D	N.D	N.D	N.D	N.D	N.D	N.D	N.D	N.D	N.D	N.D	N.D	N.D	N.D	N.D	N.D	N.D	N.D	0.00
S A4	N.D	N.D	N.D	N.D	N.D	N.D	N.D	N.D	N.D	N.D	N.D	N.D	N.D	N.D	N.D	N.D	N.D	N.D	N.D	N.D	N.D	0.00
S A5	N.D	N.D	N.D	N.D	N.D	N.D	N.D	N.D	N.D	N.D	N.D	N.D	N.D	N.D	N.D	N.D	N.D	N.D	N.D	N.D	N.D	0.00
S A6	N.D	N.D	N.D	N.D	N.D	N.D	N.D	N.D	N.D	N.D	N.D	N.D	N.D	N.D	N.D	N.D	N.D	N.D	N.D	N.D	N.D	0.00
S A7	N.D	N.D	N.D	N.D	N.D	N.D	N.D	N.D	N.D	N.D	N.D	N.D	N.D	N.D	N.D	N.D	N.D	N.D	N.D	N.D	N.D	0.00
S A8	N.D	N.D	N.D	N.D	N.D	N.D	N.D	N.D	N.D	N.D	N.D	N.D	N.D	N.D	N.D	N.D	N.D	N.D	N.D	N.D	N.D	0.00
S A9	N.D	N.D	N.D	N.D	N.D	N.D	N.D	N.D	N.D	N.D	N.D	N.D	N.D	N.D	N.D	N.D	N.D	N.D	N.D	N.D	N.D	0.00
S A10	N.D	N.D	N.D	N.D	N.D	N.D	N.D	N.D	N.D	N.D	N.D	N.D	N.D	N.D	N.D	N.D	N.D	N.D	N.D	N.D	N.D	0.00
S A11	N.D	N.D	N.D	N.D	N.D	N.D	N.D	N.D	N.D	N.D	N.D	N.D	N.D	N.D	N.D	N.D	N.D	N.D	N.D	N.D	N.D	0.00
S A12	N.D	N.D	N.D	N.D	N.D	N.D	N.D	N.D	N.D	N.D	N.D	N.D	N.D	N.D	N.D	N.D	N.D	N.D	N.D	N.D	N.D	0.00
S A13	N.D	N.D	N.D	N.D	N.D	N.D	N.D	N.D	N.D	N.D	N.D	N.D	N.D	N.D	N.D	N.D	N.D	N.D	N.D	N.D	N.D	0.00
S A14	N.D	N.D	N.D	N.D	N.D	N.D	N.D	N.D	N.D	N.D	N.D	N.D	N.D	N.D	N.D	N.D	N.D	N.D	N.D	N.D	N.D	0.00
S A15	N.D	N.D	N.D	N.D	N.D	N.D	N.D	N.D	N.D	N.D	N.D	N.D	N.D	N.D	N.D	N.D	N.D	N.D	N.D	N.D	N.D	0.00
S A16	N.D	N.D	N.D	N.D	N.D	N.D	N.D	N.D	N.D	N.D	N.D	N.D	N.D	N.D	N.D	N.D	N.D	N.D	N.D	N.D	N.D	0.00
S A17	N.D	N.D	N.D	N.D	N.D	N.D	N.D	N.D	N.D	N.D	N.D	N.D	N.D	N.D	N.D	N.D	N.D	N.D	N.D	N.D	N.D	0.00
S A18	N.D	N.D	N.D	N.D	N.D	N.D	N.D	N.D	N.D	N.D	N.D	N.D	N.D	N.D	N.D	N.D	N.D	N.D	N.D	N.D	N.D	0.00
S A19	N.D	N.D	N.D	N.D	N.D	N.D	N.D	N.D	N.D	N.D	N.D	N.D	N.D	N.D	N.D	N.D	N.D	N.D	N.D	N.D	N.D	0.00
S A20	N.D	N.D	N.D	N.D	N.D	N.D	N.D	N.D	N.D	N.D	N.D	N.D	N.D	N.D	N.D	N.D	N.D	N.D	N.D	N.D	N.D	0.00

Table S12. Fennel tea

	1	2	3	4	5	6	7	8	9	10	11	12	13	14	15	16	17	18	19	20	21	T O T A L (mg/kg)
S A1	N.D	N.D	N.D	N.D	N.D	N.D	N.D	N.D	N.D	N.D	N.D	N.D	N.D	N.D	N.D	N.D	N.D	N.D	N.D	N.D	N.D	0.00
S A2	N.D	N.D	N.D	N.D	N.D	N.D	N.D	N.D	N.D	N.D	N.D	N.D	N.D	N.D	N.D	N.D	N.D	N.D	N.D	N.D	N.D	0.00
S A3	N.D	N.D	N.D	N.D	N.D	N.D	N.D	N.D	N.D	N.D	N.D	N.D	N.D	N.D	N.D	N.D	N.D	N.D	N.D	N.D	N.D	0.00
S A4	N.D	N.D	N.D	N.D	N.D	N.D	N.D	N.D	N.D	N.D	N.D	N.D	N.D	N.D	N.D	N.D	N.D	N.D	N.D	N.D	N.D	0.00
S A5	N.D	N.D	N.D	N.D	N.D	N.D	N.D	N.D	N.D	N.D	N.D	N.D	N.D	N.D	N.D	N.D	N.D	N.D	N.D	N.D	N.D	0.00
S A6	N.D	N.D	N.D	N.D	N.D	N.D	N.D	N.D	N.D	N.D	N.D	N.D	N.D	N.D	N.D	N.D	N.D	N.D	N.D	N.D	N.D	0.00
S A7	N.D	N.D	N.D	N.D	N.D	N.D	N.D	N.D	N.D	N.D	N.D	N.D	N.D	N.D	N.D	N.D	N.D	N.D	N.D	N.D	N.D	0.00
S A8	N.D	N.D	N.D	N.D	N.D	N.D	N.D	N.D	N.D	N.D	N.D	N.D	N.D	N.D	N.D	N.D	N.D	N.D	N.D	N.D	N.D	0.00
S A9	N.D	N.D	N.D	N.D	N.D	N.D	N.D	N.D	N.D	N.D	N.D	N.D	N.D	N.D	N.D	N.D	N.D	N.D	N.D	N.D	N.D	0.00
S A10	N.D	N.D	N.D	N.D	N.D	N.D	N.D	N.D	N.D	N.D	N.D	N.D	N.D	N.D	N.D	N.D	N.D	N.D	N.D	N.D	N.D	0.00
S A11	N.D	N.D	N.D	N.D	N.D	N.D	N.D	N.D	N.D	N.D	N.D	N.D	N.D	N.D	N.D	N.D	N.D	N.D	N.D	N.D	N.D	0.00
S A12	N.D	N.D	N.D	N.D	N.D	N.D	N.D	N.D	N.D	N.D	N.D	N.D	N.D	N.D	N.D	N.D	N.D	N.D	N.D	N.D	N.D	0.00
S A13	N.D	N.D	N.D	N.D	N.D	N.D	N.D	N.D	N.D	N.D	N.D	N.D	N.D	N.D	N.D	N.D	N.D	N.D	N.D	N.D	N.D	0.00
S A14	N.D	N.D	N.D	N.D	N.D	N.D	N.D	N.D	N.D	N.D	N.D	N.D	N.D	N.D	N.D	N.D	N.D	N.D	N.D	N.D	N.D	0.00
S A15	N.D	N.D	N.D	N.D	N.D	N.D	N.D	N.D	N.D	N.D	N.D	N.D	N.D	N.D	N.D	N.D	N.D	N.D	N.D	N.D	N.D	0.00
S A16	N.D	N.D	N.D	N.D	N.D	N.D	N.D	N.D	N.D	N.D	N.D	N.D	N.D	N.D	N.D	N.D	N.D	N.D	N.D	N.D	N.D	0.00
S A17	N.D	N.D	N.D	N.D	N.D	N.D	N.D	N.D	N.D	N.D	N.D	N.D	N.D	N.D	N.D	N.D	N.D	N.D	N.D	N.D	N.D	0.00
S A18	N.D	N.D	N.D	N.D	N.D	N.D	N.D	N.D	N.D	N.D	N.D	N.D	N.D	N.D	N.D	N.D	N.D	N.D	N.D	N.D	N.D	0.00
S A19	N.D	N.D	N.D	N.D	N.D	N.D	N.D	N.D	N.D	N.D	N.D	N.D	N.D	N.D	N.D	N.D	N.D	N.D	N.D	N.D	N.D	0.00

Table S1 3. Hibiscus tea

	1	2	3	4	5	6	7	8	9	10	11	12	13	14	15	16	17	18	19	20	21	TOTAL (mg/kg)
SA1	N.D	N.D	N.D	N.D	N.D	N.D	N.D	N.D	N.D	N.D	N.D	N.D	N.D	N.D	N.D	N.D	N.D	N.D	N.D	N.D	N.D	0.00
SA2	N.D	N.D	N.D	N.D	N.D	N.D	N.D	N.D	N.D	N.D	N.D	N.D	N.D	N.D	N.D	N.D	N.D	N.D	N.D	N.D	N.D	0.00
SA3	N.D	N.D	N.D	N.D	N.D	N.D	N.D	N.D	N.D	N.D	N.D	N.D	N.D	N.D	N.D	N.D	N.D	N.D	N.D	N.D	N.D	0.00
SA4	N.D	N.D	N.D	N.D	N.D	N.D	N.D	N.D	N.D	N.D	N.D	N.D	N.D	N.D	N.D	N.D	N.D	N.D	N.D	N.D	N.D	0.00
SA5	N.D	N.D	N.D	N.D	N.D	N.D	N.D	N.D	N.D	N.D	N.D	N.D	N.D	N.D	N.D	N.D	N.D	N.D	N.D	N.D	N.D	0.00
SA6	N.D	N.D	N.D	N.D	N.D	N.D	N.D	N.D	N.D	N.D	N.D	N.D	N.D	N.D	N.D	<LOQ	N.D	N.D	N.D	N.D	N.D	0.00
SA7	N.D	N.D	N.D	N.D	N.D	N.D	N.D	N.D	N.D	N.D	N.D	N.D	N.D	N.D	N.D	N.D	N.D	N.D	N.D	N.D	N.D	0.00
SA8	N.D	N.D	N.D	N.D	N.D	N.D	N.D	N.D	N.D	N.D	N.D	N.D	N.D	N.D	N.D	N.D	N.D	N.D	N.D	N.D	N.D	0.00
SA9	N.D	N.D	N.D	N.D	N.D	N.D	N.D	N.D	N.D	N.D	N.D	N.D	N.D	N.D	N.D	N.D	N.D	N.D	N.D	N.D	N.D	0.00
SA10	N.D	N.D	N.D	N.D	N.D	N.D	N.D	N.D	N.D	N.D	N.D	N.D	N.D	N.D	N.D	N.D	N.D	N.D	N.D	N.D	N.D	0.00
SA11	N.D	N.D	N.D	N.D	N.D	N.D	N.D	N.D	N.D	N.D	N.D	N.D	N.D	N.D	N.D	<LOQ	N.D	N.D	N.D	N.D	N.D	0.00
SA12	N.D	N.D	N.D	N.D	N.D	N.D	N.D	N.D	N.D	N.D	N.D	N.D	N.D	N.D	N.D	N.D	N.D	N.D	N.D	N.D	N.D	0.00
SA13	<LOQ	N.D	N.D	N.D	N.D	N.D	N.D	N.D	N.D	N.D	N.D	N.D	N.D	N.D	N.D	N.D	N.D	N.D	N.D	N.D	N.D	0.00
SA14	N.D	N.D	N.D	N.D	N.D	N.D	N.D	N.D	N.D	N.D	N.D	N.D	N.D	N.D	N.D	N.D	N.D	N.D	N.D	N.D	N.D	0.00
SA15	N.D	N.D	N.D	N.D	N.D	N.D	N.D	N.D	N.D	N.D	N.D	N.D	N.D	N.D	N.D	N.D	N.D	N.D	N.D	N.D	N.D	0.00
SA16	N.D	N.D	N.D	N.D	N.D	N.D	N.D	N.D	N.D	N.D	N.D	N.D	N.D	N.D	N.D	N.D	N.D	N.D	N.D	N.D	N.D	0.00
SA17	<LOQ	N.D	N.D	N.D	N.D	N.D	N.D	N.D	N.D	<LOQ	N.D	N.D	N.D	N.D	N.D	N.D	N.D	N.D	N.D	N.D	N.D	0.00
SA18	N.D	N.D	N.D	N.D	N.D	N.D	N.D	N.D	N.D	N.D	N.D	N.D	N.D	N.D	N.D	N.D	N.D	N.D	N.D	N.D	N.D	0.00
SA19	N.D	N.D	N.D	N.D	N.D	N.D	N.D	N.D	N.D	N.D	N.D	N.D	N.D	N.D	N.D	N.D	N.D	N.D	N.D	N.D	N.D	0.00

Note. 1. Echimidine; 2. Heliotrine; 3. Lasiocarpine; 4. Lycopsamine; 5. Monocrotaline N oxide; 6. Monocrotaline; 7. Retrorsine N oxide; 8. Retrorsine; 9. Senecionine N oxide; 10. Senecionine; 11. Seneciphylline N oxide; 12. Seneciphylline; 13. Senkirkine; 14. Trichodesmine; 15. Europine N oxide; 16. Intermedine; 17. Jacobine; 18. Europine; 19. Jacobine N

oxide; 20. Lasiocarpine N oxide; 21. Heliotrine N oxide.
